# Prediction of incident heart failure in individuals without prior cardiovascular disease: the SCORE2-HF risk model

**DOI:** 10.1093/eurheartj/ehag154

**Published:** 2026-03-11

**Authors:** SCORE2-HF Writing Group and European Society of Cardiology’s Cardiovascular Risk Collaboration (CRC) Unit, Stephen Kaptoge, Spencer J Keene, Tessa Reitsma, Wen Shi, Jennifer S Lees, Laura Lõo, Mari Nordbø Gynnild, Chimweta Chilala, Scott Ritchie, Sarah Spackman, Gianluigi Saverese, Salil Deo, Xavier Rossello, Bin Zhou, Barry R Davis, Elizabeth L M Barr, Jarett Berry, Hermann Brenner, Edoardo Casiglia, Kevin Damman, Lori B Daniels, James de Lemos, Chiara Donfrancesco, Marcus Dörr, James S Floyd, Richard Gillum, Dominique Hange, J Wouter Jukema, Stefan Kiechl, Jorge R Kizer, Anna Kucharska-Newton, Daniel Levy, Dianna J Magliano, Kunihiro Matsushita, Kirsten Mehlig, Peter M Nilsson, Dorothea Nitsch, Børge G Nordestgaard, Michael H Olsen, Luigi Palmieri, Bruce M Psaty, Hee-Choon Shin, Lara Simpson, Johan Sundström, Jozine M Ter Maaten, Valérie Tikhonoff, Stella Trompet, Anne Tybjærg-Hansen, Henry Völzke, Kristian Wachtell, Peter Willeit, Ruijie Xie, Shuyue Yang, Gregory Y H Lip, Håvard Dalen, Juan J Carrero, Majid Ezzati, Maryam Kavousi, John William McEvoy, Massimo Piepoli, Kamlesh Khunti, Stefan Koudstaal, John Danesh, Angela Wood, Frank L J Visseren, Nathalie Conrad, Taavi Tillmann, Łukasz Kuźma, Charlotte Andersson, Steven H J Hageman, Emanuele Di Angelantonio, Lisa Pennells

**Keywords:** Heart failure, Risk prediction model, Risk estimation, Risk score, Primary prevention, CVD prevention, SCORE2 models

## Abstract

**Background and Aims:**

Heart failure (HF) presents a significant and growing public health challenge. The aim of this study was to develop and validate SCORE2-HF, a model for HF risk estimation in European adults aged over 40 years without previous cardiovascular disease.

**Methods:**

Using data from 25 prospective cohorts (14 countries, 611 778 individuals, 21 818 incident HF events), the sex-specific, competing risk-adjusted SCORE2-HF models were derived, including age, smoking status, systolic blood pressure, antihypertensive treatment, body mass index (BMI), estimated glomerular filtration rate, and type 2 diabetes mellitus (including age at diagnosis and glycated haemoglobin). Using Europe-wide statistics from the World Health Organization and linked health records from five countries (>36 million individuals, 515 466 incident HF events), models were recalibrated to contemporary 10-year and 30-year HF incidence in four European risk regions. SCORE2-HF was validated using data from three further cohorts (three countries; 1 336 824 participants; 36 841 incident HF events).

**Results:**

In the three external validation cohorts, C-indices (95% confidence interval) were .827 (.824–.829), .839 (.827–.850), and .874 (.863–.884). SCORE2-HF risks varied importantly by individual's risk factors and risk region. For example, in the low-risk region, the average 10-year risk for 70-year-old individuals with zero vs four adverse risk factors (smoking, type 2 diabetes mellitus, hypertension, and BMI ≥ 30 kg/m^2^), was 8% vs 24% in men and 6% vs 20% in women. By contrast, in the very high-risk region, the average SCORE2-HF risk with four adverse risk factors was 59% in 70-year-old men or women.

**Conclusions:**

SCORE2-HF—a model derived, recalibrated, and validated to estimate 10-year and 30-year risk of incident HF across European countries—may enhance the identification of individuals at higher risk of developing HF.

## Structured Graphical Abstract

**Structured Graphical Abstract:**
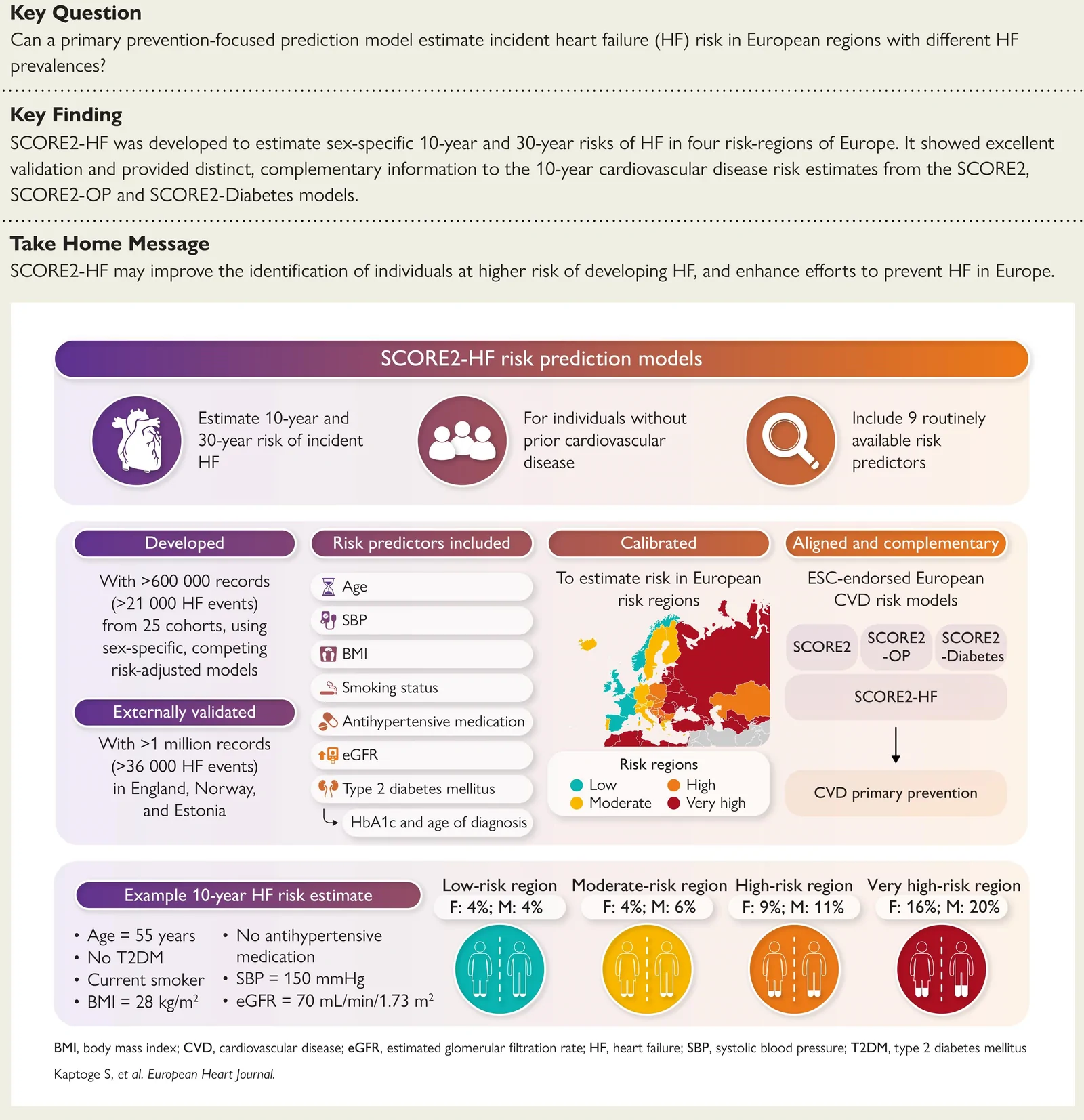

**See the editorial comment for this article ‘Risk scores for heart failure: powerful for populations, problematic for the individual patient’, by C. Torp-Pedersen**  ***et al.*****, https://doi.org/10.1093/eurheartj/ehag183.**

## Introduction

Heart failure (HF)—a condition that leads to diminished quality of life, frequent hospitalizations, substantial healthcare costs, and high mortality rates^1,2^—presents a significant and growing public health challenge.^3,4^ Globally, age-standardized prevalence has increased over the past three decades.^5^ Age-standardized rates have increased in the UK and USA in the last decade, including for individuals <70 years,^6,7^ but the overall burden is significantly propelled by population ageing.^4,8^

There may be unrealized opportunities to enhance HF prevention, linking it with established initiatives that aim to prevent atherosclerotic cardiovascular disease (CVD).^9–13^ For example, the 2021 European Society of Cardiology (ESC) guidelines on CVD prevention, the 2023 Guidelines on CVD and diabetes, and the 2025 focused update of the guidelines for management of dyslipidaemias, recommend personalized risk assessment using the SCORE2, SCORE2-OP, and SCORE2-Diabetes risk models to guide preventive interventions.^10,11,13–16^ However, while these SCORE2 models estimate 10-year risk of non-fatal myocardial infarction, non-fatal stroke, or CVD death, they do not include non-fatal HF as an outcome, highlighting opportunities for additional targeted strategies to mitigate HF risk in high-risk individuals,^17^ such as with sodium–glucose cotransporter 2 (SGLT2) inhibitors that substantially reduce HF risk in high-risk adults without established CVD.^18,19^

Most existing HF risk prediction models are not specifically focused on individuals without prior CVD, making them unsuitable for use in primary prevention strategies.^20^ Furthermore, HF models that are focused on primary prevention have limitations, especially for use in the European context, including lack of derivation or validation in European populations; inclusion of risk predictors not routinely available in general practice, e.g. electrocardiogram (ECG) variables^12,20,21^; and/or failure to statistically adapt (‘recalibrate’) models to the level of HF risk in contemporary target populations.^12,20,21^

To address these limitations, we detail the development and validation of SCORE2-HF, a new prediction model to estimate risk of incident HF among individuals aged over 40 years without previous CVD across Europe.

## Methods

### Study design

**Figure 1:**
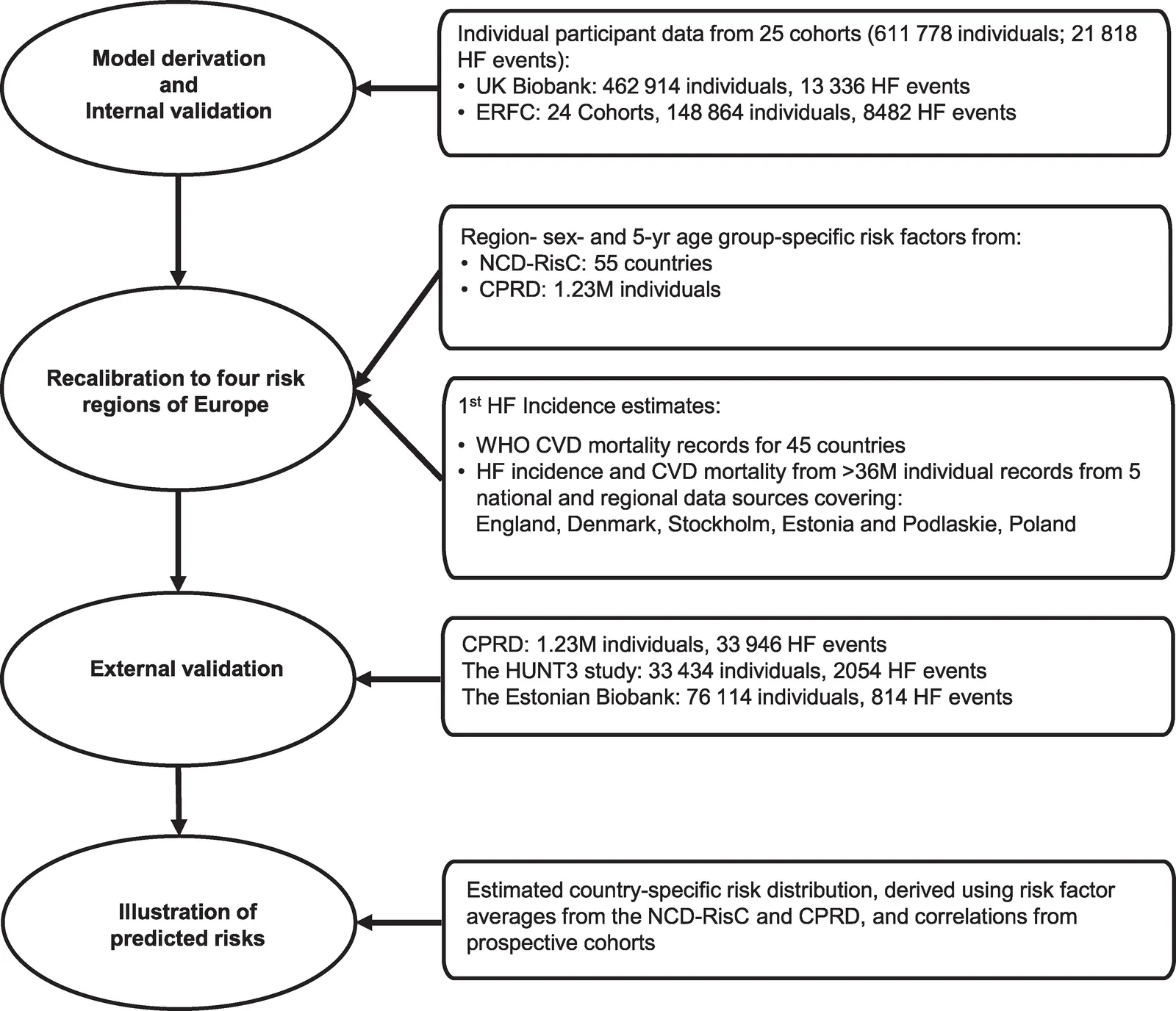
Flow diagram describing model development process and data sources. ERFC, Emerging Risk Factors Collaboration; NCD-RisC, NCD Risk Factors Collaboration; CPRD, Clinical Practice Research Datalink; WHO, World Health Organization, HUNT3, Survey 3 of the Trøndelag Health Study

The SCORE2-HF project involved multiple data sources (*Figure 1*). First, to enable reliable estimation of age- and sex-specific relative risks, we derived models for the prediction of fatal and non-fatal HF outcomes using individual-participant data from the UK Biobank (UKB) cohort^22^ and 24 cohorts from the Emerging Risk Factors Collaboration (ERFC).^23^ Second, to ensure applicability to the broader contemporary primary prevention population in different regions of Europe, we recalibrated the derived risk models using estimated age- and sex-specific incidence estimates and risk factor distributions derived from contemporary national or subnational population-representative data sources. Third, to enhance validity and generalizability, we performed external validation using individual-participant data from England's Clinical Practice Research Datalink (Gold data source, CPRD),^24^ Norway's Trøndelag Health Study (Third survey: HUNT3),^25^ and the Estonian Biobank (2018 wave, EstBB).^26^ Fourth, to illustrate the variation of CVD risk across European countries, we applied the model to contemporary populations.

### Data sources

For model derivation, we used individual-participant data from UKB and ERFC. UK Biobank is a large prospective cohort study with individual-participant data on ∼500 000 participants aged > 40 years recruited across 23 UK-based assessment centres between 2006 and 2010 and followed up for cause-specific morbidity and mortality through linkages to multiple health records.^22^ The ERFC has collated and harmonized individual-participant data from >100 long-term prospective cohort studies of CVD risk factors and outcomes.^23^ Prospective studies in the ERFC were included in this analysis if they met all the following criteria: were approximately population-based [i.e. did not select participants on the basis of having previous disease (e.g. case–control studies)]; had recorded cause-specific deaths and/or non-fatal HF events for at least 1 year of follow-up; and had recorded baseline information on the following risk factors necessary to derive risk prediction models: age, current smoking, history of type 2 diabetes mellitus (T2DM), systolic blood pressure (SBP), antihypertensive medication use, body mass index (BMI), estimated glomerular filtration rate [eGFR, estimated using the 2021 Chronic Kidney Disease Epidemiology Collaboration (CKD-EPI) creatinine-based equations^27^] or creatinine (to calculate eGFR), and T2DM characteristics [age at diagnosis and glycated haemoglobin (HbA1c), or glucose].

To match the target populations of the SCORE2, SCORE2-Diabetes, and SCORE2-OP risk scores, individuals from UKB and ERFC cohorts with and without T2DM and aged 40 years or over were included for model derivation if they had no recorded history of CVD or HF at baseline (see Supplementary data online, *Figure S1*; prior disease definitions are detailed in Supplementary data online, *Table S1*).

SCORE2-HF was recalibrated to reflect contemporary HF incidence in four risk regions of Europe using methodology described in the statistical analysis and Supplementary methods. This involved the use of summary data on risk predictors obtained from the Non-Communicable Disease Risk Factor Collaboration (NCD-RisC),^28,29^ and CPRD, and estimates of first HF incidence computed using a combination of CVD mortality rates from the World Health Organization (WHO) mortality database and national or subnational electronic health records data on >36 million individuals in five European countries. The electronic health records comprised the entire populations aged over 40 years in England (2022–24),^30^ Denmark (2010–19, 2021–22),^31^ the Stockholm region of Sweden (2010–21),^32^ Estonia (2015–23),^33^ and the Podlaskie region of Northeastern Poland (2014–15).^34^ In all data sources used for recalibration, non-fatal HF outcomes were captured using hospital admissions data with HF in any diagnostic position. Additional information from primary care records were used to exclude those with prior CVD where relevant (see Supplementary data online, *Table S1*). Further details of the five included data sources are given in Supplementary data online, *Tables S2* and *S3* and *Figure S2*.

Data from CPRD,^24^ HUNT3,^35^ and EstBB^26^ were used for external validation of SCORE2-HF. Full details of the data sources are given in Supplementary data online, *Table S4*; all represent general populations, and inclusion criteria were the same as for the cohorts used for model derivation (see Supplementary data online, *Figure S1*).

The primary outcome was fatal or non-fatal HF (with either preserved or reduced ejection fraction). Follow-up was until the first non-fatal HF hospitalization, death, or end of the registration period. Non-fatal, non-HF CVD events during follow-up were ignored. Deaths not due to HF (non-HF deaths) were treated as competing events. International Classification of Diseases 10th Revision (ICD-10) codes included in the outcomes are provided in Supplementary data online, *Table S1*. A 10-year time-frame for risk prediction was chosen to align with previous CVD risk estimation systems already in clinical use.^14–16^ In addition, 30-year risk estimation was incorporated to allow risk estimation over a longer time period for individuals aged 40–59 whose 10-year risk may appear low, despite adverse levels of risk predictors. Missing data were imputed, using multiple imputation with chained equations, as described in the Supplementary methods.

### Statistical analysis

For model derivation, sex-specific coefficients [i.e. subdistribution hazard ratios (SHRs)] were estimated using Fine and Gray competing risk-adjusted models, stratified by cohort, and where relevant, by trial arm.^36^ The sex-specific models included the following predictors: age (years), smoking (current vs other), history of T2DM (yes/no, defined by self-report and biochemical measurements/treatments), SBP (mmHg), antihypertensive medication use (current vs other), BMI (kg/m^2^), and eGFR (mL/min/1.73 m^2^). For individuals with T2DM, age at diagnosis (years) and HbA1c (mmol/mol) were also included. The risk factors were selected *a priori* due to their predictive ability based on previous literature^20,21^ as well as their availability in target populations for screening/clinical use, derivation cohorts, and population statistics needed for model recalibration. The modelling approach also aligns with the SCORE2, SCORE2-OP, and SCORE2-Diabetes models, which also comprise sex-specific Fine and Gray models with similar readily available risk factors. Since previous research showed that associations of these risk factors with HF decline with increasing age,^37^ age interactions were included for all predictors. An interaction between SBP and antihypertensive medication was also included to capture increased risk associated with treatment resistant hypertension. All continuous risk factors were included in the model without categorization. Quadratic terms were included for eGFR and BMI to parsimoniously capture non-linear associations observed in exploratory analysis of the shape of dose–response relationships using fractional polynomials. There were no (or very minimal) violations of proportional hazards assumptions, as assessed by inclusion of time varying coefficients.

Risk models were recalibrated to four risk regions using methods detailed in the Supplementary methods. Briefly, risk regions were defined to align with the SCORE2 project (low-, moderate-, high-, and very-high-risk regions of Europe defined by their CVD mortality rates; Supplementary data online, *Table S5*). For each risk region, expected HF risk was compared to predicted SCORE2-HF risk using sex-specific linear regressions of the transformed risks over 5-year age groups. The intercept and slope from the regression were applied to recalibrate the models. This process was completed for recalibration of the ERFC/UKB models to give both 10-year risks and 30-year risks.

For the ‘x-axis’ values in the recalibration regressions, predicted risks were estimated by applying the uncalibrated SCORE2-HF risk models to sex and age group-specific average risk factor values from each risk region, obtained from the NCD-RisC collaboration^28,29^ (supplemented by CPRD estimates for eGFR, HbA1c, and age of diabetes diagnosis only).

For the ‘y-axis’ values in the recalibration regressions, expected 10-year and 30-year HF risks, adjusted for competing risk of non-HF death, were estimated by sex and age group for each risk region using a lifetable approach. The lifetable was constructed from derived estimates of HF incidence and non-HF mortality, which were obtained by applying multipliers, pooled from the five representative data sources (see Supplementary data online, *Tables S2* and *S3*), to the region, sex, and age group-specific WHO CVD mortality and non-HF mortality estimates. The multiplier's calculation formula was designed to ensure that the resulting rates applied to the population with no prior CVD or HF. In this way, absolute HF risk for individual countries was determined primarily according to the country's regional CVD mortality rate and the ratio of HF incidence to CVD mortality, which was consistently observed across the five representative data sources. Full details of the methods used to estimate and apply the multipliers are given in the Supplementary methods.

For validation, we assessed discrimination using Harrell's C-index, utilizing all available follow-up time and adjusting for competing risks.^38^ For internal validation, the C-index was calculated separately within each of the ERFC/UKB cohorts and pooled, weighted by the number of contributing events,^39^ and 10-fold cross-validation was performed to check for optimism. Calibration was assessed by graphical comparison of predicted and observed risks (with adjustment for competing risk). The performance of SCORE2-HF was also compared with that seen for the AHA-PREVENT-HF, using the published, simplified logistic regression implementation of PREVENT-HF.^12^

To compare the proportion of the population estimated to be at different levels of HF risk according to the SCORE2-HF models, predicted risk distributions were simulated using age- and sex-specific risk factor value means and prevalences from NCD-RisC and correlation structures observed in ERFC cohorts.

## Results

Model derivation involved a total of 611 778 individuals without previous CVD from UKB and 24 ERFC cohorts (*Table 1*; Supplementary data online, *Table S6*). The median [interquartile range (IQR)] age at recruitment was 58 years (49–64) among men and 58 years (51–64) among women (*Table 1*), with men constituting 45% of the cohort (275 006 individuals).

**Table 1:** Summary of data used for derivation of the sex-specific SCORE2-HF models

	Men *N* = 275 006 (45%)	Women *N* = 336 772 (55%)
Predictors included in the risk model		
Age (years)	58 [49–64]	58 [51–64]
Systolic blood pressure (mmHg)	140 (18)	136 (20)
Body mass index (kg/m^2^)	27.5 (4.2)	27.1 (5.3)
eGFR (mL/min/1.73 m^2^)	95 [83–103]	94.6 [80–102]
Current hypertensive medication use	61 229 (23%)	68 903 (21%)
Current smoking	48 481 (18%)	38 481 (11%)
History of type 2 diabetes	21 657 (8%)	19 140 (6%)
Predictors used only in those with diabetes		
HbA1c (mmol/mol)	51 [41–64]	52 [42–66]
Age of diabetes diagnosis (years)	54 [46–60]	55 [46–61]
Incident events and follow-up		
Heart failure	12 201 (4%)	9617 (3%)
Non-heart failure mortality	23 821 (9%)	21 170 (6%)
Follow-up (years)	13.4 [12.3–14.2]	13.4 [12.4–14.2]

Data are presented as median [interquartile range], *n* (%), or mean (standard deviation).

eGFR, estimated glomerular filtration rate (estimated using the 2021 Chronic Kidney Disease Epidemiology Collaboration creatinine-based equations); HbA1c, glycated haemoglobin.

**Figure 2:**
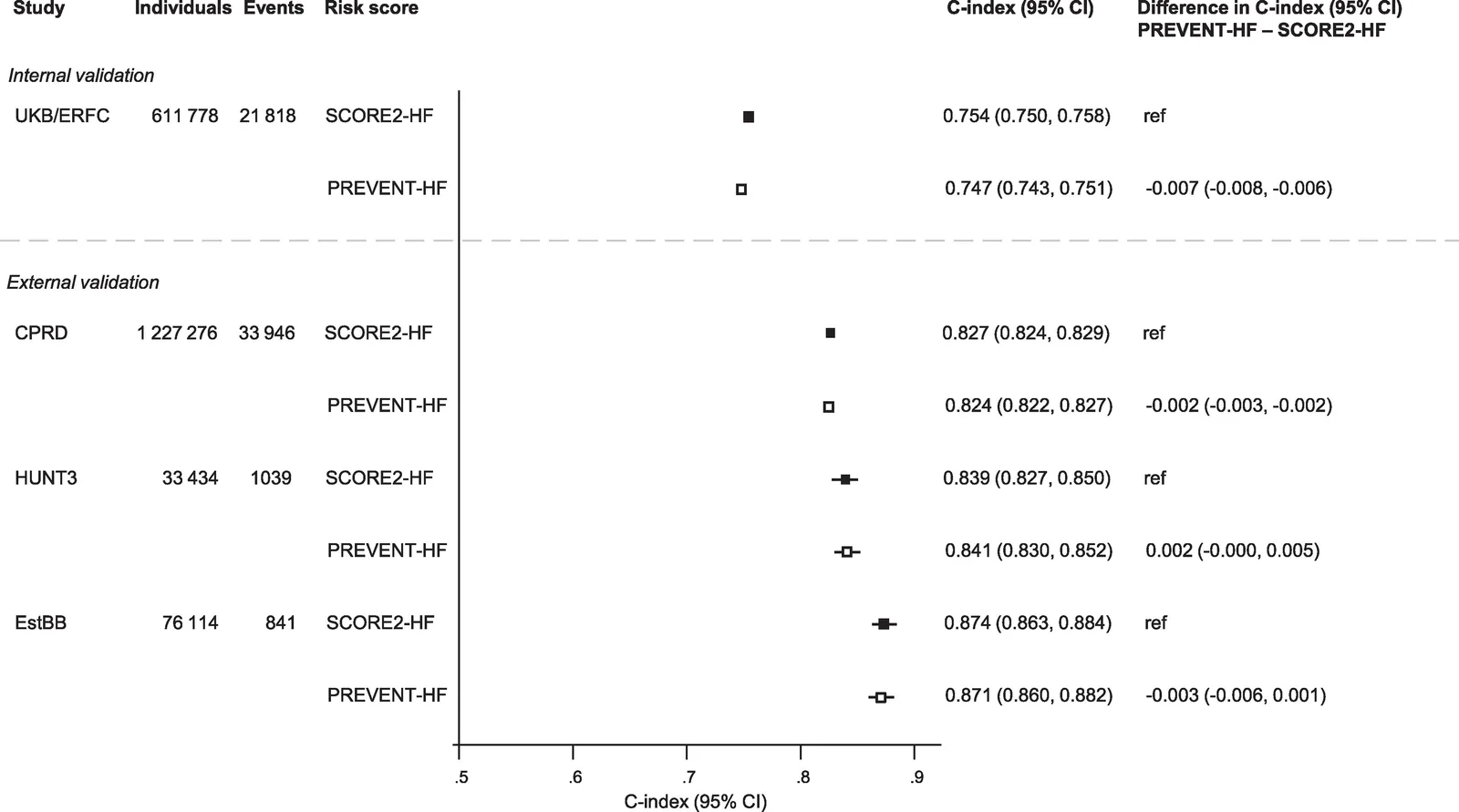
C-index results from internal and external validation. CPRD, Clinical Practice Research Datalink; HUNT3, Survey 3 of the Trøndelag Health Study; EstBB, Estonian Biobank; UKB, UK Biobank; ERFC, Emerging Risk Factors Collaboration. For internal validation, 10-fold cross validation revealed minimal optimism in discrimination (optimism in C-index = 0.0003 (95% CI = 0.0002, 0.0003); Supplementary methods)

At a median (IQR) follow-up of 13.4 (12.3–14.2) years, a total of 21 818 first HF events were recorded [12 201 (56%) among men]. Subdistribution hazard ratios are shown in Supplementary data online, *Table S7*. The magnitude of relative associations of model predictors generally decreased with increasing age of participants (see Supplementary data online, *Figure S3*). Competing risk-adjusted associations of smoking, T2DM, and antihypertensive medication use with HF were greater in magnitude in women than in men. The SCORE2-HF models showed good discrimination in the derivation data (*Figure 2*) with a C-index [95% confidence interval (CI)] of .754 (.750–.758).

For recalibration, estimation of sex- and age-specific first HF incidence rates involved the application of multipliers from five data sources across low-, moderate-, and high-risk regions (see Supplementary data online, *Table S3* and *Figure S4*). Multipliers were similar in different sources and so were pooled to give sex and age group-specific values for use across all risk regions. Multipliers were applied, within each risk region, to WHO CVD mortality statistics, to estimate first HF incidence, and to WHO non-HF mortality statistics to adjust these to be relevant to the population with no prior CVD (see Supplementary methods). After application of the multipliers, the estimated first HF incidence rates increased with age and risk region and were similar in men and women (see Supplementary data online, *Figure S5*). After recalibration using the estimated recalibration factors (see Supplementary data online, *Table S8*), the SCORE2-HF predicted risks based on mean risk factor levels showed good agreement with the estimated HF event incidence (see Supplementary data online, *Figure S5*).

SCORE2-HF was externally validated using data from 1 227 276 individuals (33 946 HF events) from CPRD, 33 434 individuals (2054 HF events) from HUNT3, and 76 114 individuals (841 HF events) from EstBB, without previous CVD (see Supplementary data online, *Table S9*). SCORE2-HF showed good discrimination, with a C-index (95% CI) of .827 (.825–.829) in CRPD, .839 (.827–.850) in HUNT3, and .874 (.863–.884) in EstBB (*Figure 2*), which was similar to discrimination seen with the AHA PREVENT-HF risk score [C-index = .824 (.822–.827) in CPRD, .841 (.830–.852) in HUNT3, and .871 (.860–.882) in EstBB]. C-indices were slightly higher in external validation cohorts since these had a wider age range than most cohorts used in the derivation datasets^39^ (see Supplementary data online, *Tables S6* and *S9*).

Good discrimination was seen across various subgroups of the population, including those defined by sex, age, and T2DM and was comparable to PREVENT-HF in all of these subgroups (see Supplementary data online, *Figures S6*–*S8*). Good discrimination was also seen for the 30-year SCORE2-HF models over the available follow-up period (around 10, 16, and 5 years for CPRD, HUNT3, and EstBB, respectively), and this was similar to that seen with PREVENT-HF 30-year risk models (see Supplementary data online, *Figure S9*). SCORE2-HF was generally well calibrated in the contemporary and nationally representative data from CPRD, showing slight overestimation of risk in those under the age of 70 years. Miscalibration was slightly greater for PREVENT-HF, which overestimated risk for men and women of all ages (see Supplementary data online, *Figure S10*). Likewise, SCORE2-HF showed good calibration specifically among individuals with T2DM from CPRD (see Supplementary data online, *Figure S11*).

**Figure 3:**
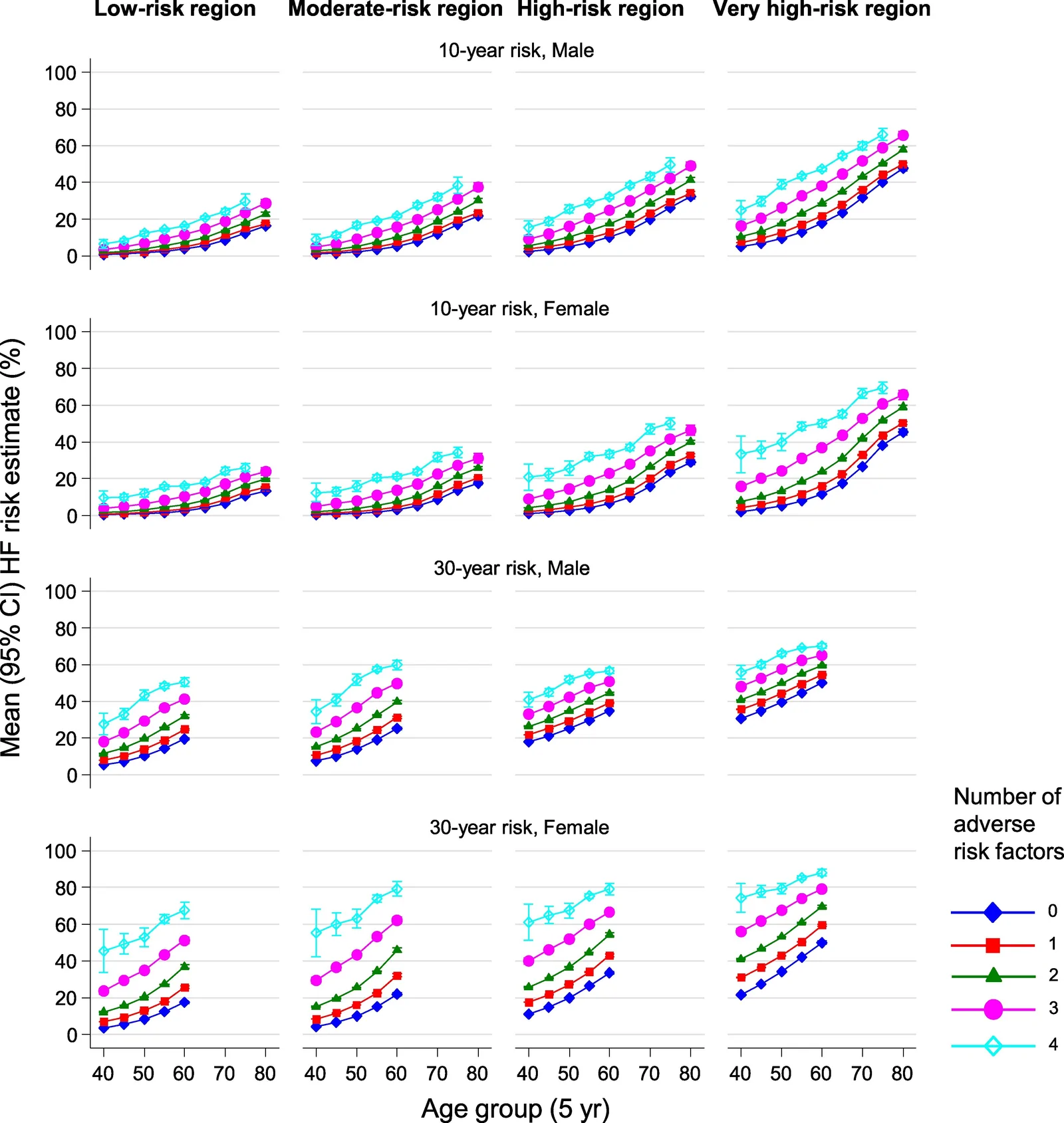
Average SCORE2-HF risk estimates for individuals with 0, 1, 2, 3 or 4 adverse heart failure risk factors, by risk region, sex and age group. Adverse risk factors are: smoking, type 2 diabetes, hypertension (defined as taking antihypertensive medication or systolic blood pressure ≥ 140 mmHg) or body mass index ≥ 30 kg/m^2^. Each line illustrates the *average* predicted risk among sex- and age group-specific individuals who have been categorized according to the given definitions of adverse risk factors. However, individual risk estimates within each category depend on the full continuous range of systolic blood pressure, body mass index, estimated glomerular filtration rate and glycated haemoglobin. Individual-specific risks can be estimated using the calculator provided in Supplementary data online, Appendix, and similar electronic calculators or apps, all of which utilise the full range of continuous risk factors. CI, confidence interval, HF, heart failure

Risk estimations varied importantly across individuals with different risk predictor values (*Figure 3*). For example, among 70-year-old men or women from the low-risk region who were non-smokers, with no history of T2DM, hypertension (defined as antihypertensive use or SBP > 140 mmHg) or obesity (defined as BMI > 30 kg/m^2^), the average 10-year risk of HF was estimated to be 8% and 6%, respectively. However, for 70-year-old men or women from the low-risk region who were current smokers, with T2DM, hypertension, and obesity, the average estimated 10-year risk of HF was 24% and 20%, respectively. Risk also increased substantially with risk region, with an average estimated 10-year risk for 70-year-old men or women with none of these adverse risk factors reaching 30% and 26%, respectively, in the very high-risk region (around four-fold higher compared with similar individuals in the low-risk region). Likewise, for 70-year-olds in the very high-risk region with all four adverse risk factor values, average risks were 59% for both men and women. The pattern of 30-year risks across individuals and risk regions was similar to that seen with 10-year risks (*Figure 3*). To facilitate use of SCORE2-HF during clinical encounters, a calculator to estimate individual 10-year or 30-year risk is provided in Supplementary data online, Appendix  *S1*, using the process exemplified in Supplementary data online, *Supplementary methods Table 1*.

**Figure 4:**
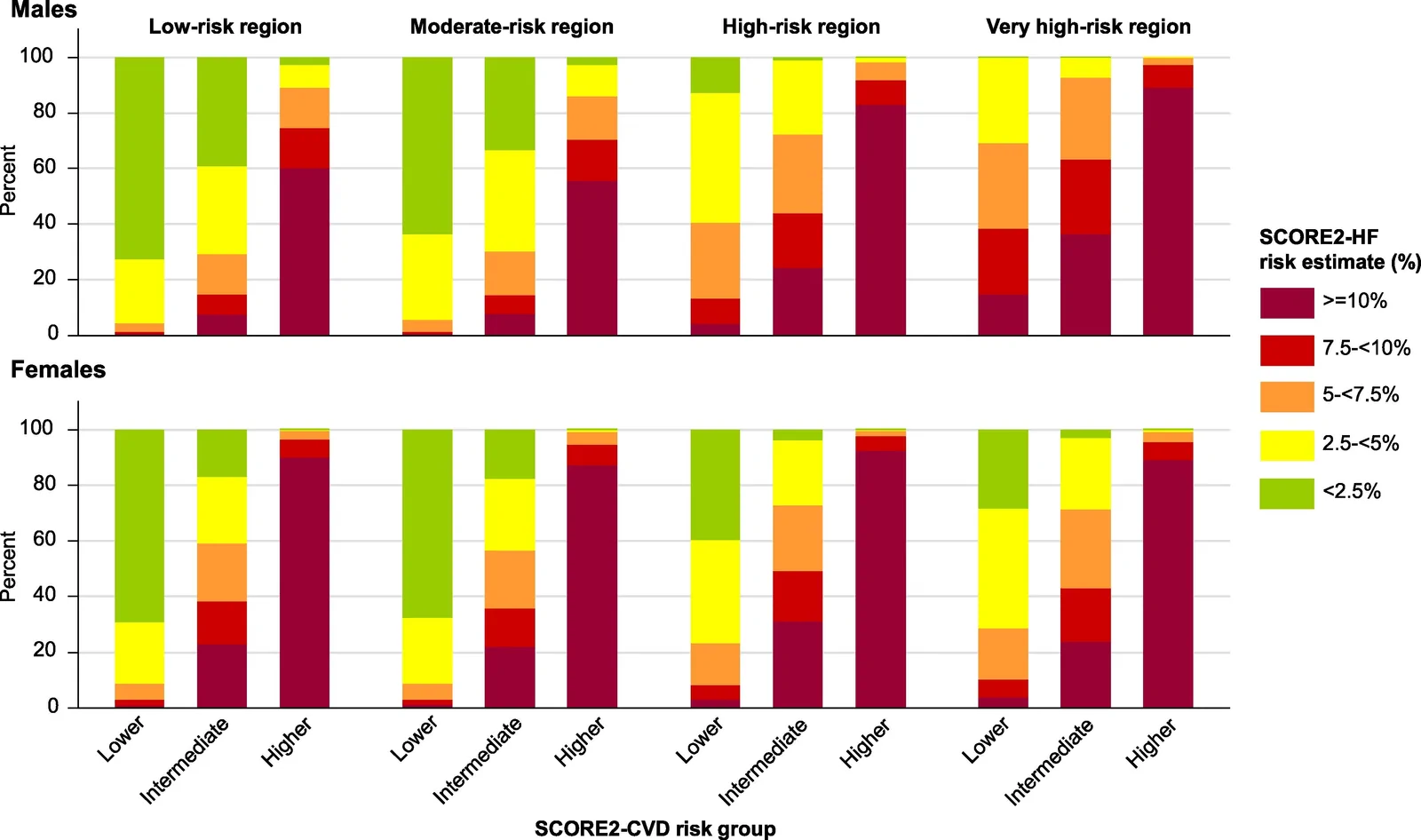
Comparison of risk classification with SCORE2 and SCORE2-HF. SCORE2-CVD risk groups are determined according to ESC guidelines on cardiovascular disease (CVD) prevention (2021) and CVD and diabetes (2023). In the 2021 guidelines, the ‘intermediate risk group’ is defined as ‘high-risk’ and includes those aged 40 to 49 years at 2.5 to <7.5% 10-year CVD risk, aged 50 to 69 at 5 to <10% risk and those aged 70 years or over at 7.5 to <15% risk. According to the guideline, this high-risk group identifies individuals in whom ‘risk factor treatment should be considered’ and the very high-risk group as those in whom ‘risk factor treatment is generally recommended’. In the 2023 guidelines on CVD and diabetes, the ‘intermediate’ risk group is defined as ‘moderate risk’ and includes individuals with diabetes, with 10-year risk of 5 to <10%. Predicted risk distributions are based on the observed risk factor distributions in individuals from the CPRD

Analysis using the individual participant risk factor distributions observed in CPRD suggested that SCORE2-HF provided further informative risk stratifications within CVD risk categories using the SCORE2-CVD risk algorithm as recommended by the ESC guidelines (*Figure 4*). For example, the 2021 guidelines for CVD prevention^10^ recommend ‘consideration of risk factor treatment’ among ‘high-risk’ individuals without diabetes according to age-specific SCORE2-CVD risk thresholds (10-year risks of 2.5%–7.5% for those aged 40–49, 5%–10% for those aged 50–69, and 7.5%–15% for those age ≥70). Likewise, the 2023 CVD and diabetes guidelines^11^ recommend that individuals with diabetes and 10-year CVD risk between 5% and 10% should be classified as ‘moderate-risk’. We jointly termed such defined ‘high’ or ’moderate’ risk individuals as ‘intermediate’ risk in our analysis. Using these thresholds, 59%, 55%, 46%, and 27% of men were defined as intermediate risk in the low-, moderate-, high-, and very-high-risk regions of Europe, respectively. Of these men, 6%, 8%, 25%, and 38% were estimated to have ≥10% risk of incident HF. Likewise for the 32%, 35%, 31%, and 30% of women classified at ‘intermediate’ CVD risk according to the guideline CVD risk thresholds, 22%, 21%, 31%, and 24% would be at >10% risk of incident HF according to SCORE2-HF.

**Figure 5:**
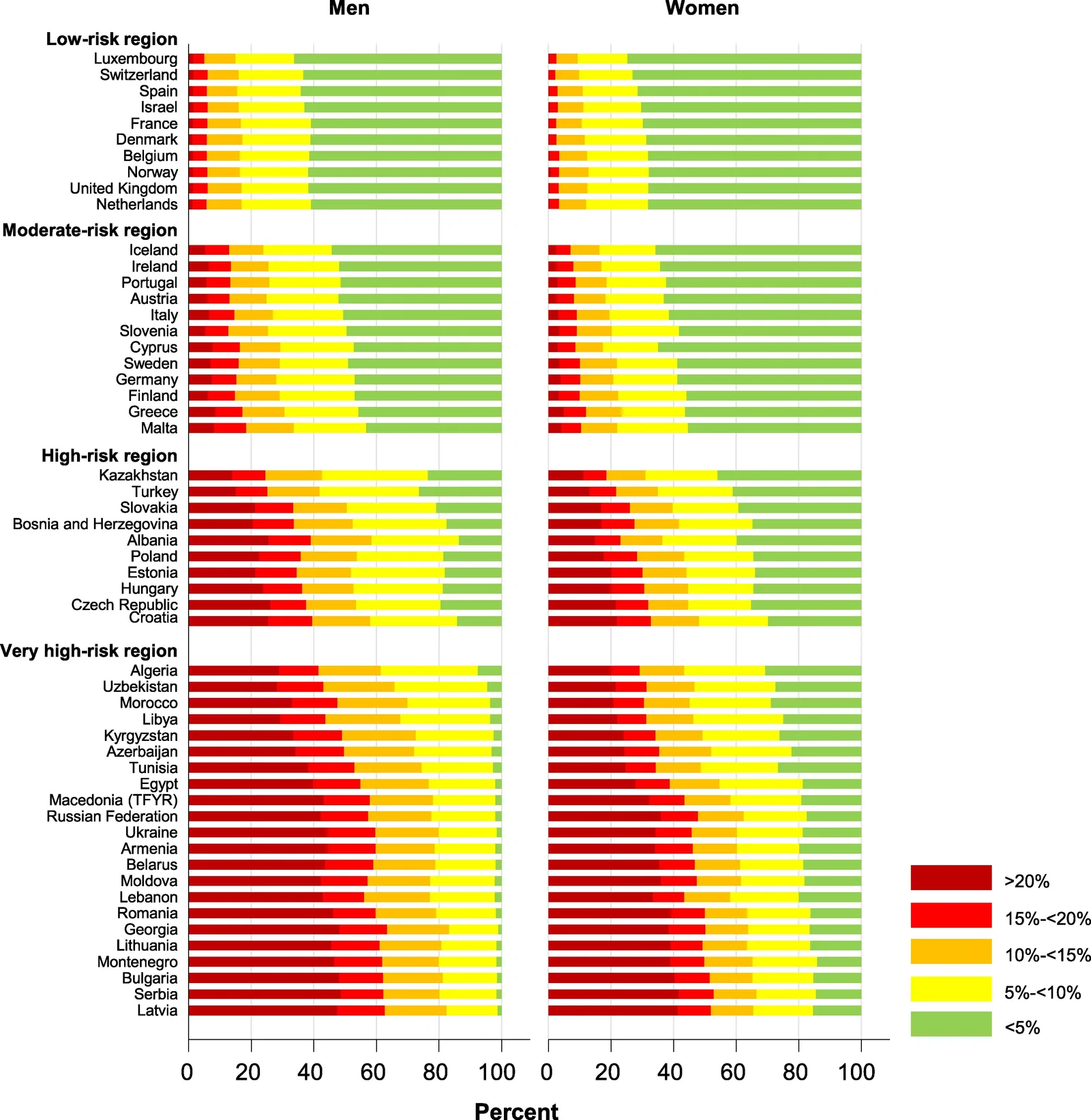
Estimated risk distributions using SCORE2-HF in individual countries across Europe. Predicted risk distributions were simulated using age- and sex-specific risk factor value means and prevalences from NCD-RisC and correlation structures observed in cohorts from the Emerging Risk Factors Collaboration

The distribution of SCORE2-HF risk estimates varied importantly across populations from different European countries (*Figure 5*), with over 80% of men and over 85% of women expected to be at <10% 10-year HF risk in most countries in the low-risk regions but fewer than 30% of men and fewer than 50% of women at <10% risk in the very high-risk region. As expected, HF risk estimates increased steeply with age in all risk regions (see Supplementary data online, *Figure S12*).

## Discussion

To help empower HF prevention across European populations, we have developed, validated, and illustrated SCORE2-HF, an algorithm designed to estimate the 10-year and 30-year risks of incident HF in individuals aged over 40 years, with or without T2DM, and with no prior history of CVD or HF. Designed to complement existing SCORE2 algorithms and to maximize clinical uptake, SCORE2-HF incorporates information routinely available for risk estimation in primary care settings. As SCORE2-HF has several advantages over previously developed HF risk prediction algorithms, it could enhance the identification of individuals at high HF risk across Europe who might benefit most from preventive interventions (*Structured Graphical Abstract*).

First, SCORE2-HF has been recalibrated to the HF risk observed in the broad target primary prevention populations across four risk regions of Europe. By contrast, some previous HF models were derived in cohort studies not representative of the broader contemporary target populations in which they are intended for use. Whereas previous models have been liable to systematic mis-estimation of risk^40^ due to their failure to account for such variations in risks by data source or geographical location, SCORE2-HF has limited such biases through systematic recalibration.

Second, the recalibration approach used in SCORE2-HF has been designed so that models can be readily updated to reflect future HF incidences and risk factor profiles of any target population of apparently healthy individuals who need risk assessment. Importantly, the model's updating approach is pragmatic, cost-effective, and scalable, requiring only contemporary aggregate-level data concerning the target population.

Third, as this approach to recalibration involves the same methods and European risk region definitions as used in other models recommended by the ESC (e.g. SCORE2, SCORE2-OP, and SCORE2-Diabetes), it means that CVD and HF risk estimation could be readily synergized across Europe, empowering their concurrent primary prevention. Furthermore, compared to risk predictors used in SCORE2 risk estimation, SCORE2-HF additionally requires only eGFR, BMI, and antihypertensive medication use allowing for easy simultaneous risk estimation. We have provided an excel calculator for estimation of SCORE-HF 10-year and 30-year risks (see Supplementary data online, *Appendix S1*) and further online calculators/apps will be made available to facilitate risk estimation with both the SCORE2/SCORE2-OP/SCORE2-Diabetes models and SCORE2-HF.

Fourth, we have also demonstrated that SCORE2-HF provides both distinct and complementary information to the SCORE2 models, giving a more complete picture of the total CVD risk faced by individuals. For example, the 2021 ESC guidelines on CVD prevention recommend that individuals identified by SCORE2 to be in the age-specific ‘high-risk’ category should *consider* medical intervention (primarily to lower lipids and/or blood pressure) in a shared decision-making process. In a representative population, we have shown a broad range of SCORE2-HF risk estimates among individuals in this subgroup, which could be used to inform such decisions. Likewise, for individuals with T2DM and no prior CVD, the decision to initiate glucose-lowering medication (e.g. SGLT2 inhibitors) is currently influenced by SCORE2-Diabetes estimates,^11^ but could also take into account SCORE2-HF estimates. SCORE2-HF was designed to estimate HF risk in the primary prevention context, thereby completing the suite of SCORE2 models for CVD prevention in the general population. Other risk prediction tools provide HF risk estimation in the secondary prevention context, including SMART2-HF^41^ for individuals with existing CVD and LIFE-Preserved^42^ and LIFE-HF^43^ for individuals with a history of HF.

Fifth, SCORE2-HF includess a facility to estimate 30-year HF risk in individuals aged under 60 years, enabling estimation of longer-term risk in younger individuals with substantial risk factor burden, whose 10-year risk may, nonetheless, appear modest.

Sixth, we have shown that SCORE2-HF performs well in external validation, with good discrimination and calibration. Discrimination was similar to that seen with the AHA-PREVENT-HF risk models, but the systematic recalibration of SCORE2-HF ensured it performed better in terms of calibration in the low-risk region and is also likely to be better calibrated to moderate-, high-, and very-high-risk countries.

Finally, our project has illustrated the expected risk classification of individuals using SCORE2-HF, using data estimated from all European countries, showing that the proportions of individuals in specific risk categories differ substantially across countries. This diversity highlights why policymakers and practitioners need tailored tools such as SCORE2-HF to help make more appropriate and locally informed decisions about the allocation of prevention resources.

The potential limitations of this effort merit consideration. We derived the risk prediction models in predominantly low-risk cohorts from 15 countries (with 78% of contributing individuals from the UK). Ideally, however, the derivation of risk models would have involved large nationally representative cohorts across all four European risk regions. Unfortunately, availability of such data across Europe is limited. Indeed, even the European cohorts included may not be nationally representative, reflecting a baseline from past decades and self-selected participants such as healthy volunteers.^44^ However, while healthy volunteer bias can lead to low estimates of absolute risk, relative risks are generally unaffected.^45^ Our approach therefore makes the assumption that the relative risks obtained in the derivation dataset are transferable across different populations, and this is evidenced by good discrimination of the score in external validation across three countries.

Furthermore, to avoid systematically inaccurate absolute risk estimation due to the biases of cohort studies, we recalibrated models using nationally representative HF incidence rates. Currently SCORE2-HF has been recalibrated using WHO CVD mortality data from all European risk regions, adapted to represent HF incidence in those with no prior CVD, using relationships observed between CVD mortality and HF incidence in five large national or sub-national data sources. In the future, should reliable data on first event HF incidence become available by region, country, or within-country region, they can be used to refine the recalibration of SCORE2-HF.

To quantify HF rates in the data sources used for recalibration, non-fatal incident HF was ascertained from hospital admission data and therefore may not capture chronic HF diagnoses managed only in outpatient settings. Furthermore, underdiagnosis of HF has been previously observed in both primary and secondary care settings.^46,47^ However, our use of hospital admissions records with HF in any diagnostic position (rather than primary position only) is likely to recover some HF diagnoses made (or missed) earlier in outpatient settings. While identification of individual HF cases using any vs primary position has been shown to have slightly lower positive predictive value, the aggregate level incidence by sex and age group used in SCORE2-HF recalibration may be better captured using this approach.^48^ Likewise, while the diagnostic validity of the first HF diagnosis appearing on a death certificate may be questionable,^49^ this category accounted for <1% of observed HF incidence used for recalibration. Heart failure incidence was therefore captured as accurately and consistently as possible given current data availability across Europe, and the recalibration approach outlined can be applied in the future, should more reliable data on HF incidence become available in nationally representative European primary care populations. None of these concerns are relevant for the estimation of the relative risk estimates for SCORE2-HF individual predictors, since model derivation was based predominantly on individual participant cohort data with validated endpoints. Further splitting of incidence rates by HF type was not possible; SCORE2-HF estimates the risk of any HF type, without discrimination between preserved or reduced left ventricular ejection fraction.

It should be noted that SCORE2-HF estimates HF risk regardless of other CVD events that may occur in the interim; this is aligned with previous risk scores^50^ and ensures relevance of estimates for assignment of interventions that focus on HF prevention. However, given that some HF events will occur subsequent to another CVD event (this occurred in 8% of the HF events observed in our CPRD validation dataset), simple summation of SCORE2 CVD and HF risk estimates to give total CVD risk is not advisable and may result in inflated estimates of overall CVD risk.

There could be some individuals who contributed data to more than one of the UK studies analysed (UKB, CPRD, and NHS England-wide EHR). However, given that data from these sources were collected over different time periods (median baseline was 2009 for UKB, 2006 for CPRD, and 2022 for NHS England) using distinct protocols/methods, any precise data overlap is presumed minor and unlikely to bias our validation in CPRD. Some individuals in our model derivation cohorts may have initiated preventative treatment (e.g. statins) during follow-up, and accounting for this could improve model calibration and discrimination. However, previous analyses have suggested that inclusion of information on statin initiation during follow-up provides only limited clinical and public health benefit.^51^

Data on the type of antihypertensive medication use, family history, ethnicity, socio-economic status, physical activity, smoking duration and/or former smoking, atrial fibrillation, or breathing disorders (e.g. asthma and chronic obstructive pulmonary disease) were either not consistently available or not uniformly defined across the data sources used for model derivation and recalibration. Hence, interpretation of SCORE2-HF estimates may require clinical judgement for individuals in whom these factors are relevant. Furthermore, while SCORE2-HF captures HF risk associated with kidney function through inclusion of eGFR, additional assessment of albuminuria may add complementary information for understanding individual kidney disease and HF risk.^10,11,52^ Likewise, several cardiac biomarkers not included in SCORE2-HF have been demonstrated to improve accuracy of risk estimates for HF and may warrant measurement in those found to be at intermediate risk.^53–55^

In conclusion, we have developed, recalibrated, and validated SCORE2-HF, a HF risk score tailored to the levels of HF risk in the primary prevention population in four European risk regions, and aligned with the SCORE2 models. SCORE2-HF estimations rely on routinely available risk predictors and should enhance efforts to prevent CVD and HF development in Europe.

## Supplementary Material

ehag154_Supplementary_Data

## Declarations

### Data Availability

Data used for the current study are available upon reasonable request and approval of the individual cohorts or collaborative groups.

## Appendix

### Authors from the SCORE2-HF working group

Stephen Kaptoge^1,2^*, Spencer J. Keene^1,2^*, Tessa Reitsma^3^*, Wen Shi^1,2^*, Jennifer S. Lees^4^*, Laura Lõo^5^, Mari Nordbø Gynnild^6,7,8^, Chimweta Chilala^1,2^, Scott Ritchie^1,2,9,10,11^, Sarah Spackman^1,2^, Gianluigi Saverese^12^, Salil Deo^13,14,15^, Xavier Rossello^16^, Bin Zhou^17^, Barry R Davis^18^, Elizabeth L.M. Barr^19,20^, Jarett Berry^21^, Hermann Brenner^22,23^, Edoardo Casiglia^24^, Kevin Damman^25^, Lori B. Daniels^26^, James de Lemos^21^, Chiara Donfrancesco^27^, Marcus Dörr^28,29^, James S. Floyd^30^, Richard Gillum^31^, Dominique Hange^32^, J. Wouter Jukema^33,34^, Stefan Kiechl^35^, Jorge R. Kizer^36^, Anna Kucharska-Newton^37^, Daniel Levy^38^, Dianna J. Magliano^39^, Kunihiro Matsushita^40^, Kirsten Mehlig^32^, Peter M. Nilsson^41,^, Dorothea Nitsch^42^, Børge G. Nordestgaard^43,44^, Michael H. Olsen^44,45^, Luigi Palmieri^27^, Bruce M. Psaty^30^, Hee-Choon Shin^46^, Lara Simpson^18^, Johan Sundström^47,48^, Jozine M. Ter Maaten^25^, Valérie Tikhonoff^49^, Stella Trompet^33,50^, Anne Tybjærg-Hansen^43,44^, Henry Völzke^28,29^, Kristian Wachtell^51^, Peter Willeit^1,52,53^, Ruijie Xie^54,55^, Shuyue Yang^56^, Gregory Y. H. Lip^,57,58,59^, Håvard Dalen^6,7,60^, Juan J Carrero^61^, Majid Ezzati^17^, Maryam Kavousi^56^, John William McEvoy^62^, Massimo Piepoli^63,64^, Kamlesh Khunti^65^, Stefan Koudstaal^66^, John Danesh^1,2,9,67,68,69^, Angela Wood^1,2,9,67,70^, Frank L. J. Visseren^3^, Nathalie Conrad^71,72^, Taavi Tillmann^5^, Łukasz Kuźma^59,73,74,^ Jannick A. N. Dorresteijn^3^**, Charlotte Andersson^75,76^**, Steven H. J. Hageman^3^**, Emanuele Di Angelantonio^1,2,67,68,77^**, Lisa Pennells^1,2,9^**^§^

*Contributed equally

**Contributed equally

^§^Corresponding author: lamp2@medschl.cam.ac.uk (L.P.)

**Affiliations**

1. British Heart Foundation Cardiovascular Epidemiology Unit, Department of Public Health and Primary Care, University of Cambridge, Papworth Road, Cambridge Biomedical Campus, Cambridge, UK.
2. Victor Phillip Dahdaleh Heart and Lung Research Institute, University of Cambridge, Cambridge UK.
3. Department of Vascular Medicine and Endocrinology, University Medical Centre Utrecht, Utrecht, The Netherlands
4. School of Cardiovascular & Metabolic Health, University of Glasgow, Glasgow, UK
5. Institute of Family Medicine and Public Health, University of Tartu, Tartu, Estonia
6. Clinic of Cardiology, St. Olav University Hospital, Trondheim, Norway.
7. Department of Circulation and Medical Imaging, Faculty of Medicine and Health Science, NTNU—Norwegian, University of Science and Technology, Trondheim, Norway.
8. K.G. Jebsen Center for Cardiac Biomarkers, Institute of Clinical Medicine, University of Oslo, Oslo, Norway
9. British Heart Foundation Centre of Research Excellence, University of Cambridge, Cambridge, UK
10. Cambridge Baker Systems Genomics Initiative, Department of Public Health and Primary Care, University of Cambridge, Cambridge, UK
11. Cambridge Baker Systems Genomics Initiative, Baker Heart & Diabetes Institute, Melbourne, Victoria, Australia
12. Department of Medicine, Karolinska Institutet, Stockholm, Sweden
13. Surgical Services, Louis Stokes Cleveland VA Medical Center, Cleveland, OH, USA.
14. Case School of Medicine, Case Western Reserve University School of Medicine, Cleveland, OH, USA.
15. School of Health and Wellbeing, University of Glasgow, Glasgow, UK.
16. Hospital Universitario Son Espases, Health Research Institute of the Balearic Islands, Universitat Illes Balears, Palma de Mallorca, Spain
17. MRC Centre for Environment and Health, Department of Epidemiology and Biostatistics, School of Public Health, Imperial College London, London, UK
18. University of Texas School of Public Health, Houston, TX, USA.
19. Clinical Diabetes and Epidemiology, Baker Heart and Diabetes Institute, Melbourne, Australia
20. Menzies School of Health Research, Charles Darwin University, Darwin, Northern Territory, Australia
21. Department of Internal Medicine, The University of Texas Southwestern Medical Center, Dallas, TX, USA.
22. Cancer Prevention Graduate School, German Cancer Research Center (DKFZ), Heidelberg, Germany.
23. Network Aging Research, Heidelberg University, Heidelberg, Germany
24. Department of Medicine, Studium Patavinum, University of Padua, Padua, Italy
25. Department of Cardiology, University Medical Center Groningen, Groningen, The Netherlands.
26. Sulpizio Cardiovascular Center, Department of Medicine, University of California San Diego, La Jolla, CA, USA
27. Department of Cardiovascular, Endocrine-Metabolic Diseases and Aging, Istituto Superiore di Sanita, Rome, Italy
28. German Centre for Cardiovascular Disease (DZHK), Partner Site Greifswald, Germany.
29. University Medicine Greifswald, Greifswald, Germany
30. Cardiovascular Health Research Unit, Departments of Medicine and Epidemiology, University of Washington, Seattle, WA, USA
31. Department of Medicine, Howard University College of Medicine, Washington, DC, USA
32. School of Public Health and Community Medicine/Primary Health Care, Institute of Medicine, Sahlgrenska Academy, University of Gothenburg, Gothenburg, Sweden.
33. Department of Cardiology, Leiden University Medical Center, Leiden, The Netherlands.
34. Netherlands Heart Institute, Utrecht, The Netherlands
35. Department of Neurology and VASCage, Medical University Innsbruck, Innsbruck, Austria
36. Cardiology Section, San Francisco Veterans Affairs Health Care System, and Departments of Medicine, Epidemiology and Biostatistics, University of California San Francisco, San Francisco, CA, USA
37. Department of Epidemiology, Gillings School of Global Public Health, University of North Carolina at Chapel Hill, Chapel Hill, NC, USA
38. National Heart Lung and Blood Institute, Framingham, MA, USA
39. Diabetes and Population Health, Baker Heart and Diabetes Institute, Melbourne, Australia
40. Bloomberg School of Public Health, Johns Hopkins University, Baltimore, MD, USA
41. Lund University, Department of Clinical Sciences, Malmö, Sweden
42. Faculty of Epidemiology and Population Health, The London School of Hygiene & Tropical Medicine, London, UK
43. Copenhagen University Hospital, Copenhagen, Denmark
44. Department of Clinical Medicine, University of Copenhagen, Copenhagen, Denmark.
45. Department of Internal Medicine 1, Holbaek Hospital, Holbaek, Denmark
46. Independent researcher, Naperville, IL, USA
47. Clinical Epidemiology Unit, Department of Medical Sciences, Uppsala University, Uppsala, Sweden.
48. The George Institute for Global Health, University of New South Wales, Sydney, Australia.
49. Department of Medicine—DIMED, University of Padua, Padua, Italy
50. Department of Internal Medicine, section of Gerontology and Geriatrics, Leiden University Medical Center, Leiden, The Netherlands
51. Department of Cardiology, Odense University Hospital, Denmark
52. Institute of Clinical Epidemiology, Public Health, Health Economics, Medical Statistics and Informatics, Medical University of Innsbruck, Innsbruck, Austria.
53. Ignaz Semmelweis Institute, Interuniversity Institute for Infection Research, Medical University of Vienna, Vienna, Austria
54. Division of Clinical Epidemiology of Early Cancer Detection, German Cancer Research Center (DKFZ), Heidelberg, Germany
55. Medical Faculty, Heidelberg University, Heidelberg, Germany
56. Department of Epidemiology, Erasmus MC, University Medical Centre Rotterdam, Rotterdam, The Netherlands
57. Liverpool Centre for Cardiovascular Science at University of Liverpool, Liverpool John Moores University and Liverpool Heart & Chest Hospital, Liverpool, UK
58. Danish Center for Health Services Research, Department of Clinical Medicine, Aalborg University, Aalborg, Denmark
59. Medical University of Bialystok, Department of Cardiology, Lipidology and Internal Medicine with Intensive Coronary Care Unit, Białystok, Poland
60. Levanger Hospital, Nord-Trøndelag Hospital Trust, Levanger, Norway
61. Department of Medical Epidemiology and Biostatistics, Karolinska Institutet, Stockholm, Sweden
62. Department of Cardiology, Erasmus Medical Centre Rotterdam, The Netherlands, University of Galway School of Medicine and National Institute for Prevention and Cardiovascular Health, Galway, Ireland.
63. University Cardiology Department, IRCCS Policlinico San Donato, San Donato Milanese, Milan, Italy
64. Department of Biomedical Sciences for Health, University of Milan, Milan, Italy
65. Diabetes Research Centre, The University of Leicester, Leicester, UK
66. Department of Cardiology, Erasmus Medical Centre Rotterdam, Rotterdam, The Netherlands.
67. Health Data Research UK Cambridge, Wellcome Genome Campus and University of Cambridge, Cambridge, UK
68. National Institute for Health and Care Research Blood and Transplant Research Unit in Donor Health and Behaviour, University of Cambridge, Cambridge, UK
69. Department of Human Genetics, the Wellcome Trust Sanger Institute, Wellcome Trust Genome Campus, Hinxton, UK
70. British Heart Foundation Data Science Centre, Health Data Research UK, London, UK
71. Nuffield Department of Women's and Reproductive Health, University of Oxford, UK
72. Department of Cardiovascular Sciences, Leuven, Belgium
73. Department of Invasive Cardiology, Medical University of Białystok, Białystok, Poland
74. Department of Cardiac Surgery and Transplantology, National Medical Institute of the Ministry of the Interior and Administration, Warsaw, Poland
75. Department of Cardiology, Copenhagen University Hospital Herlev and Gentofte, Herlev, Denmark.
76. Section of Advanced Heart Disease, Cardiology Division, Brigham & Women's hospital, Boston, MA, USA
77. Health Data Science Research Centre, Human Technopole, Milan, Italy

### ESC cardiovascular risk collaboration

Prof. Emanuele Angelantonio (Department of Public Health and Primary Care, University of Cambridge, Victor Phillip Dahdaleh Heart & Lung Research Institute), Prof. Ana Abreu (Hospital de Santa Maria, Faculty of Medicine, Lisbon), Prof. Frank Visseren (Department of Vascular Medicine, University Medical Center Utrecht), Prof. Maryam Kavousi (Department of Epidemiology, Erasmus MC, University Medical Center, Rotterdam), Prof. John William McEvoy (Preventive Cardiology, School of Medicine, University of Galway), Prof. Angela Wood (Department of Public Health and Primary Care, University of Cambridge, Victor Phillip Dahdaleh Heart & Lung Research Institute).

### Study investigators from the Emerging Risk Factors Collaboration (ERFC)

**ALLHAT:** Barry R. Davis, Lara Simpson; **ARIC:** Patricia Chang, Christie Ballantyne, Jeremy Van'T Hof; **AUSDIAB:** Jonathan Shaw, Elizabeth LM Barr, Dianna Magliano; **BRUN:** Gregorio Rungger, Karin Willeit, Johann Willeit; **CASTEL:** Edoardo Casiglia, Valérie Tikhonoff; **CHS:** Bruce M Psaty, Jorge Kizer, James Floyd; **COPEN:** Marianne Benn, Peter Schnohr, Shoaib Afzal; **DHS:** Colby Ayers, Amil Shah, Amit Khera; **ESTHER:** Bernd Holleczek, Ben Schöttker, Lei Peng; **GOTOW:** Cecilia Björkelund, Lauren Lissner, Valter Sundh; **LIFE:** Michael Hecht Olsen, Kristian Wachtell**; MATISS:** Cinzia Lo Noce, Benedetta Marcozzi, Alessandra Cardinale; **MPP:** Peter M Nilsson; **MRCOLD:** Dorothea Nitsch; **NHANESIII:** Hee-Choon Shin, Richard Gillum; **PREVEND:** Peter van de Meer, Stephan Bakker, Ron T. Gansevoort; **PROSPER:** J.Wouter Jukema, Naveed Sattar, Stella Trompet; **RANCHO:** Lori B Daniels; **ROTT:** Maryam Kavousi, Shuyue Yang; **ULSAM:** Johan Sundström.

**Study Investigator from the HUNT3 study:** Torbjørn Omland
