# Supplementary material for: Prediction of incident heart failure in individuals without prior cardiovascular disease: the SCORE2-HF risk model
Source: Eur Heart J. 2026 Mar 11;47(34):4758–72. doi: 10.1093/eurheartj/ehag154 (PMC13549722; doi:10.1093/eurheartj/ehag154)
Supplement: Supplementary file 1 [file ehag154_supplementary_data.zip › Supplementary Material.pdf]

Supplementary Material

Prediction of incident heart failure in individuals without prior cardiovascular disease: the SCORE2-HF model

Supplementary Methods

Model development and internal validation

Model derivation involved analysis of data from 611,778 individuals from 24 cohort studies (The UK Biobank and 23 studies from the Emerging Risk Factors Collaboration **Supplementary Table 6**). All datasets used were harmonised to ensure risk predictors were defined similarly, and as follows:

age (years), smoking (current vs other), history of T2DM (yes/no, defined by self-report and biochemical measurements/treatments), systolic blood pressure (mmHg), antihypertensive medication use (current vs other), BMI (kg/m^2), and eGFR ($mL/min/1.73^2$, estimated using the 2021 CKD EPI creatinine-based equations), and for individuals with T2DM, age-at- diagnosis (years) and HbA1c (mmol/mol). To estimate associations between each risk predictor and the HF outcome, sex-specific Fine and Grey models were fit, with stratification by cohort and, where relevant for 4 cohorts, by trial arm. This approach allowed for separate estimation of competing risk adjusted baseline survival estimate for each strata, but a common estimation of each sub-distribution hazard ratio across all strata (equivalent to pooling of cohort-specific SHRs using inverse variance weighted fixed effects meta-analysis). The resulting common SHRs for the sex-specific models are given in **Supplementary Methods Table 1** and **Supplementary Table 7**).

Heterogeneity in SHRs was tested using a Cox model fitted within study (application of the Fine and Gray model by study in all imputed datasets was computationally challenging due to computational intensity and high levels of non-convergence). I^2 values were calculated to estimate the amount of variation in hazard ratios that was due to between study variation, and revealed minimal concern about the generalisability of effect estimates. All values of I^2 (95% CI) were found to be substantially <50% for almost all predictors, except for the following among men: SBP (67 (54, 76)) and $eGFR^2$ (51 (29, 67)) and the following among women: Age (56 (37, 69)), BMI (51 (30, 66)) and BMI2 (58 (40, 70)). Since recalibration adapts the effect of age to the relevant risk region, heterogeneity in age coefficients was deemed to be of little concern.

During internal validation, discrimination was assessed by calculation of the C-index, adjusted for the competing risk of non-HF death (ref). C-indices were calculated within each study before pooling weighting by the number of contributing events. Since the model derivation dataset was of substantial size, little optimism due to overfitting was expected; however to check for optimism in predictive performance, 10-fold cross

validation was completed, revealing minimal evidence of optimism in the C-index, with cross validated values lower by -0.0003 (95% CI = -0.0003, -0.0002) compared to the C-index obtained without cross validation.

Recalibration methods

SCORE2-HF was recalibrated to the expected level of 10-year and 30-year HF risk in each risk region of Europe: Low, Moderate, High and Very High. This involved the following steps for each of the sex-specific models:

1) Countries were in divided into European risk regions according to the SCORE2 model categorisation based on CVD mortality (**Supplementary Table 5**)
2) Predicted risk using the uncalibrated core UKB/ERFC risk model was estimated for each 5-year age group using the median age-specific risk factor values across all risk region-specific countries, obtained from the NCD-RisC collaboration (supplemented by CPRD for eGFR, Age of diabetes diagnosis and HbA1c only).
3) Expected 1-year HF and non-HF mortality risks for each age group were estimated by combining WHO CVD mortality, averaged over countries within each risk region, with multipliers estimated in five large population data sources (see **Supplementary Methods Figure** for details of multiplier estimation and application; see **Supplementary Tables 2-3** for details of data sources used)
4) Expected 1-year HF and non-HF mortality risks were combined in a life table approach to give expected 10-year and 30-year risks for each risk region
5) Transformed expected 10-year and 30-year risk was regressed across age groups on that predicted by the core UKB/ERFC models to derive recalibration factors (the intercept and slope of the resulting regression line). The SCORE2-HF risk models were rescaled using the estimated recalibration factors.

The recalibration regression model was defined as follows:

$$\ln(-\ln(1 - \widehat{p_{expected}}(10))) = \alpha + \beta\ln(-\ln(1 - \widehat{p_{pred}}(10)))$$

Where

$\widehat{p_{expected}}(10) =$ the expected 10-year or 30-year HF event probability for each age group

$\widehat{p_{predicted}}(10) =$ the predicted 10-year HF event probability for each age group using the uncalibrated UKB/ERFC model

The estimated intercept and slope (**Supplementary Table 8**) were then used in model recalibration to give the recalibrated 10-year HF event probability, $\widehat{p_{recalbrated}}(10)$, as follows, where $\widehat{\alpha_{t10}}$ and $\widehat{\beta_{t10}}$ are the intercept and slope respectively for the 10-year risk model and $\widehat{\alpha_{t10}}$ and $\widehat{\beta_{t1}}$ for the 30 year risk model.

$$\widehat{p_{recalbrated}}(10) = 1 - exp\,(-exp\,(\widehat{\alpha_{t10}} + \widehat{\beta_{t10}}ln\,(-ln\,(1 - \widehat{p_{pred}}(10)))))$$

$$\widehat{p_{recalbrated}}(30) = 1 - exp\,(-exp\,(\widehat{\alpha_{t30}} + \widehat{\beta_{t30}}ln\,(-ln\,(1 - \widehat{p_{pred}}(10)))))$$

Supplementary Methods Table 1 shows how to make estimates for men or women from each risk region using the recalibrated model

Methods for imputation of missing data

Missing data in the derivation and validation datasets is summarised in **Supplementary Methods Table 2.**

Missing data in the model derivation dataset were imputed separately by sex using multiple imputation with chained equations (MICE) repeated five times. Continuous variables were imputed by predictive mean matching (PMM) while categorical variables were imputed using multinomial or binary logistic regression. The imputation model included all risk predictors as well as Nelson-Aalen estimates of the cumulative hazard of both HF and non-HF death outcomes by cohort and sex and indicators of each outcome. Data on outcomes, age and sex were complete. Interactions between age and the Nelson-Aalen cumulative hazard and outcome indicators were included to capture age-dependent hazard ratios of risk factors included in the model. For example, the imputation model for continuous covariate x_j was

$$x_j = \beta_0 + \beta_1 * age + \beta_2 * NA1 + \beta_3 * age * NA1 + \beta_4 * outc1 + \beta_5 * age * outc1 + \beta_6 * NA2 + \beta_7 * age * NA2 + \beta_8 * outc2 + \beta_9 * age * outc2 + \boldsymbol{\gamma X} + \in_j$$

allowing imputed values of x_j to depend on age, outcomes (i.e. Nelson-Aalen cumulative hazards and outcomes indicators), other covariates, and interactions of age and the outcomes, thereby being congenial with the inclusion of age*covariate interactions in the prediction model. Age of diabetes diagnosis was imputed conditional on history of diabetes.

In addition to the specific model risk predictors, glucose was included as an additional auxiliary variable that was more complete across cohorts to aid imputation of glycated haemoglobin (HbA1c) that was missing completely for some studies (**Supplementary Table 6**). For predictors that were entirely missing in a derivation cohort, imputation was performed by leveraging the observed values of the same predictor from other cohorts. To evaluate the validity of this cross-cohort imputation, we conducted a simulation in which all original values of a certain predictor within a cohort were temporarily set to missing. The distribution of the imputed values was then compared with the original observed values to assess how well the cross-cohort imputation performed and was found to be similar, and to result in hazard ratios.

Missing predictor values in the external validation datasets were imputed similarly (but with 10 imputations), and independently within each dataset. All risk predictors were at least partially available in each validation dataset (**Supplementary Table 6**) and there was therefore no need for inclusion of glucose as an auxiliary variable.

Supplementary Methods Table 1. Calculation of 10- and 30-year heart failure (HF) risk using SCORE2-HF

1) Calculation of Linear Predictor

Risk factor (units)	Transformed Risk factor	Log SHR Men	Log SHR Women
Risk factors applied to everyone			
Age (yrs)	cage = (age - 60)/5	0.4578	0.5298
Smoking (current vs. other)	smallbin	0.4956	0.7219
Antihypertensive medication	hypdbin	0.4629	0.5420
SBP (mm Hg)	csbp = (sbp - 120)/20	0.1456	0.1460
BMI (kg/m^2)	cbmi = (bmi – 25)/5	0.3065	0.3100
BMI^2	cbmi*cbmi	0.0027	-0.0058
Diabetes (yes vs. no)	hxdiabbin	0.1493	0.2690
Ln eGFR (ml/min/1.73m^2)	clnegfr=(lnegfr - 4.5)/0.15	-0.0298	-0.0528
Ln eGFR^2	clnegfr* clnegfr	0.0046	0.0045
Antihypertensive medication use interaction with SBP	csbp*hypdbin	-0.0443	-0.0511
Smoking interaction with age	cage*smallbin	-0.0624	-0.1064
SBP interaction with age	cage*csbp	-0.0227	-0.0096
Diabetes interaction with age	cage*hxdiabbin	0.0342	0.0149
Antihypertensive medication use interaction with age	cage*chypdbin	-0.0333	-0.0514
BMI interaction with age	cage*cbmi	-0.0154	-0.0257
Ln eGFR interaction with age	cage*clnegfr	0.0031	0.0034
Risk factors applied only in individuals with T2DM			
Diabetes age at diagnosis (yrs)	cagediab=(agediab - 50)/5*	-0.0258	-0.0921
HbA1c (mmol/mol)	chba1c=(hba1c - 31)/9.34*	0.1058	0.1182
HbA1c interaction with age	cage*chba1c	-0.0175	-0.0113

Linear predictor= ∑ (transformed risk factor x log SHR)

2) 10-year risk estimation (un-calibrated) = 1-basesurv $^{\exp(\text{linear predictor})}$

Men	Women
Uncalibrated risk = 1-0.9885 $^{\exp(\text{linear predictor})}$	Uncalibrated risk = 1-0.9924 $^{\exp(\text{linear predictor})}$

3) Calibration of risk estimate according to region specific scaling factors

Calibrated X-year risk = 1-exp(-exp(scale1 + scale2 x ln(-ln(1-un-calibrated 10-yr risk))

Risk region	Time horizon	Men	Women
**Low**	**10-year**	1-exp(-exp(0.2247+0.8758x ln(-ln(1 un-calibrated 10-yr risk))))	1-exp(-exp(-0.0229+0.8181x ln(-ln(1- un-calibrated 10-yr risk))))
	**30-year**	1-exp(-exp(1.2857+0.6971x ln(-ln(1 un-calibrated 10-yr risk))))	1-exp(-exp(1.6742+0.7311x ln(-ln(1- un-calibrated 10-yr risk))))
**Moderate**	**10-year**	1-exp(-exp(0.5441+0.8695x ln(-ln(1- un-calibrated 10-yr risk))))	1-exp(-exp(0.3562+0.8511x ln(-ln(1- un-calibrated 10-yr risk))))
	**30-year**	1-exp(-exp(1.5053+0.6786 ln(-ln(1- un-calibrated 10-yr risk))))	1-exp(-exp(2.1173+0.7724x ln(-ln(1- un-calibrated 10-yr risk))))
**High**	**10-year**	1-exp(-exp(0.7070+0.7344x ln(-ln(1- un-calibrated 10-yr risk))))	1-exp(-exp(0.7611+0.7779x ln(-ln(1 un-calibrated 10-yr risk))))
	**30-year**	1-exp(-exp(0.7495+0.3944x ln(-ln(1- un- calibrated 10-yr risk))))	1-exp(-exp(1.6327+0.5536x ln(-ln(1- un-calibrated 10-yr risk))))
**Very high**	**10-year**	1-exp(-exp(1.0927+0.6785 x ln(-ln(1- un-calibrated 10-yr risk))))	1-exp(-exp(1.2640+0.7459x ln(-ln(1- un-calibrated 10-yr risk))))
	**30-year**	1-exp(-exp(0.9650+0.3275x ln(-ln(1- un-calibrated 10-yr risk))))	1-exp(-exp(1.7426+0.4598x ln(-ln(1- un-calibrated 10-yr risk))))

Note: final estimate should be multiplied by 100 to express as a percentage.

* if hxdiabbin=1, else 0. eGFR: estimated Glomerular Filtration Rate (ml/min/1.73m^2) calculated using the CKD epi 2021 equations; HbA1c (mmol/mol): glycated haemoglobin, in International Federation of Clinical Chemistry and Laboratory Medicine (IFCC) units

Supplementary Methods Table 2. Summary of missing data in the cohort studies used for model derivation and external validation

	Derivation cohorts	Validation cohorts		
	611,778 total participants from UKB and 24 ERFC	1,227,276 patients from CPRD	33,434 participants from HUNT3	76,114 participants from Estonian Biobank
Sex	0 (0%)	0 (0%)	0 (0%)	0 (0%)
Age at survey (years)	0 (0%)	0 (0%)	0 (0%)	0 (0%)
Smoking status	3406 (0.6%)	909460 (74%)	0 (0%)	0 (0%)
Anti-hypertensives drug status	15436 (3%)	0 (0%)	4 (0.01%)	0 (0%)
SBP (mmHg)	3350 (0.5%)	504727 (41%)	208 (1%)	18,537 (24%)
BMI (kg/m2)	6233 (1%)	953006 (78%)	205 (1%)	273 (0%)
eGFR by CKDEPI 2021 (ml/min/1.73m^2)	55013 (9%)	777347 (63%)	398 (1%)	50,559 (66%)
History of diabetes	2773 (0.5%)	0 (0%)	0 (0%)	0 (0%)
Predictors used only in those with diabetes				
HbA1c (mmol/mol)) in those with diabetes	21475 (53%)	5173 (8.1%)	729 (47%)	1845 (40%)
Age at diagnosis of diabetes (years)	19939 (49%)	0 (0%)	223 (14%)	0 (0%)
Auxiliary variables used only in imputation				
Glucose* (mmol/l)	85870 (14%)	Not applicable	*Not applicable*	*Not applicable*

UKB= UK Biobank, ERFC= Emerging Risk Factors Collaboration; CPDR= Clinical Practice Research Datalink; HUNT3 = Survey 3 of The Trøndelag Health Study; EstBB= Estonian Biobank

*Glucose was used only as auxiliary variable for conditionally imputing HbA1c in the derivation cohorts. HbA1c was completely missing in 16 of the derivation cohorts, but all cohorts had glucose measurements. In the 9 cohorts that recorded HbA1c from the development samples, only 11% of individuals with diabetes had missing values.

Supplementary Methods Figure. Estimation of multipliers and application to give expected 10-year and 30-year risk of HF in each risk region of Europe

In national or regional EHR:

1) Estimate 1-year HF 'incidence' using individuals with no prior CVD or HF
2) Estimate 1-year CVD mortality using all individuals

Multiplier A =

$$\frac{HF\ incidence\ (primary\ prevention\ population)}{CVD\ mortality\ (whole\ population)}$$

3) Estimate 1-year non-HF mortality using individuals with no prior CVD or HF
4) Estimate 1-year non-HF mortality using all individuals

Multiplier B =

$$\frac{Non\text{–}HF\ mortality\ (primary\ prevention\ population)}{Non\text{–}HF\ mortality\ (whole\ population)}$$

Using WHO mortality statistics:

1) Estimate median CVD mortality for whole low risk region
2) Apply **multiplier A** to get **1st HF incidence** (primary prevention population)

3) Estimate median non-HF mortality for the whole low risk region
4) Apply **multiplier B** to get **non-HF mortality** (primary prevention population)

Combine **1 yr HF incidence** with **non-HF mortality** in a life table approach to estimate **10-year or 30-year risk of 1st HF event,** adjusted for competing risk of non-HF death

Supplementary Tables and Figures

Supplementary Table 1: Endpoints used in exclusion criteria and endpoint definition

Definition of history of HF or CVD at baseline (used to exclude participants)

Prior disease	ICD-10 code	ICD9-code
Coronary heart disease	I20-I25	410 - 414
Stroke	I60-69	430-438
TIA	G45	435.9
Peripheral artery disease	I70-71	440.2
Heart failure	I50, I11.0, I13.0, I13.2, I25.5, I42, I43, J81, K76.1, R57	428, 425

ICD codes were used to identify history of disease from hospital admissions statistics. Additional events recorded in primary care were identified using equivalent codes

Definition of the HF endpoint, either fatal or non-fatal

Endpoint	ICD-10 code	ICD9-code
Heart failure	I50*,	428
Hypertensive heart disease with (congestive) heart failure	I11.0	
Hypertensive heart and renal disease with (congestive) heart failure	I13.0	
Hypertensive heart and renal disease with both (congestive) heart failure and renal failure	I13.2	
Ischaemic cardiomyopathy	I25.5	
Dilated cardiomyopathy	I42.0	
Cardiomyopathy, unspecified	142.3, I42.5, I42.8, I42.9, I43	425
Pulmonary oedema	J81	
Chronic passive congestion of liver	K76.1	
Shock, not elsewhere classified	R57	

Disease endpoints were identified from hospital admissions statistics using the listed ICE codes only in any diagnostic position. Deaths were those identified using the underlying cause.

Supplementary Table 2: Description of the data sources used for multiplier calculation

Data source	Country	Description of data source
**Data sources used for multiplier calculation**		
NHS England	England	England-wide statistics were based on findings from the British Heart Foundation Data Science Centre CVD-COVID-UK/COVID-IMPACT Consortium (project CCU072: Burden of cardiovascular diseases in England), which involved the linkage of data records from primary care (the General Practice Extraction Service Data for Pandemic Planning and Research [GDPPR]), secondary care (Hospital Episode Statistics [HES]), the Office for National Statistics (ONS) Civil Registration of Deaths. Incident non-fatal events were obtained from HES (recorded in any diagnostic position) and deaths from ONS data where HF was the underlying cause. Information on prior/prevalent events were supplemented by primary care records, using SNOMED codes.Rates observed during the period January 2022 to December 2024 were used to ensure relevance to contemporary and post-COVID populations and avoid any instability observed during different phases of COVID-19 infections, lockdowns and vaccination (https://bhf-dsc-hds.shinyapps.io/ccu072/).
National Danish healthcare registry	Denmark	Data were from The Danish National Patient Registry, in which all contacts with the healthcare system including hospitalisation and mortality, are tracked using a personal ID number. Hospitals are reimbursed based on correct reporting of diagnoses, which ensures a high accuracy of most diagnoses. Mortality data were derived from death certificates (causes of death registry), which treating physicians are required to fill out on all individuals in Denmark. In accordance with the WHO definition only codes for 'underlying cause of death' were considered. Our study included all Danish individuals >18 years of age during the study period (from 2010 – 2022, excluding 2020).
SCREAM project	Sweden	Data were from The Stockholm CREAtinine Measurements (SCREAM) project, which contains deterministic linkage of healthcare utilisation data from all residents of the Stockholm region (Sweden). SCREAM contains complete information on demographics, healthcare use, laboratory tests, dispensed drugs, diagnoses, and vital status. Data used were from individuals aged 20 or older, from 2010 to 2021.
BIG-HEART database	Estonia	The BIG-HEART database was drawn from the population registry, including all residents aged 36 and older who lived in Estonia on 01.01.2012. Participants records were linked to health and social data from eight national registries through pseudo-IDs, facilitating analyses of healthcare utilisation, diagnoses, drug prescriptions and purchases, and a rich set of socioeconomic indicators. Annual health and mortality outcomes were linked and analysed until 31.12.2023 in this study, including all primary and secondary care across both outpatients and inpatients.
EP-PARTICLES study	Poland	The EP-PARTICLES study is a population-based, linked electronic health-record cohort from the Podlaskie Voivodeship in North Eastern Poland. Within this project, the EP-PARTICLES-HF sub-cohort was defined. Eligibility required age ≥30 years at baseline. Hospitalizations were identified from Podlaskie Voivodeship hospital discharge records and the National Health Fund (Regional Office in Białystok). Cause-specific mortality was obtained from Statistics Poland (Olsztyn Regional Statistical Office). The analytic period was 2014–2015.

Supplementary Table 3: Detailed characteristics of the data sources used for multiplier calculation (annual average for the years covered)

Country	Data source	Type of data	Area covered	Years covered	Annual average statistics						Period total events		
					Number of participants	Age Mean (SD)	Men N (%)	CVD deaths	HF events in CVD- free sub-population	HF deaths in CVD-free sub-population	CVD deaths	HF events in CVD-free sub-population	HF deaths in CVD-free sub-population
UK	NHS England	National linked Electronic Health Records	England	2022-2024	30,861,163	60.2 (13.3)	15,296,584 (50%)	105,323	97,805	2,713	315,970	293,415	8,140
Denmark	National Danish healthcare registry	National linked Electronic Health Records	Denmark	2010-2019, 2021-2022	3,226,471	59.6 (12.9)	1,587,379 (49%)	11,178	12,814	417	134,140	153,770	5,000
Sweden	SCREAM project	Regional Electronic Health data	Region of Stockholm, Sweden	2010-2021	1,226,052	60.1 (14.3)	595,902 (49%)	4,705	2,821	239	56,461	33,851	2,848
Estonia	BIG-HEART database	National linked Electronic Health Records	Estonia	2015-2023	656,672	62.7 (13.0)	289,060 (44%)	6,925	2,500	406	62,323	22,496	3,655
Poland	EP-PARTICLES study	Regional Linked Electronic Health Records	Podlaskie, North Eastern Poland	2014-2015	585,657	59.7 (12.8)	269,027 (46%)	5,177	5,967	310	10,353	11,934	620
TOTAL				2010-2024	36,556,015	60.2 (13.3)	18037952 (49%)	133,308	121,907	4,085	579,247	515,466	20,263

*Around 20% of 1st HF events were based on hospital records with HF in the primary diagnostic positions, and less than 1% were due to HF as an underlying cause of death. The remainder were from hospital records with diagnosis codes in secondary positions.

Supplementary Table 4. Description of the data sources used for model validation

**Data sources used for model validation**		
The Clinical Practice Research datalink (CPRD)[1]	UK	CPRD is an ongoing primary care database of anonymized medical records from general practitioners, with coverage of approximately 6.9% of the UK population. Included patients are broadly representative of the UK general population in terms of age, sex and ethnicity. The data used for this study is restricted to the region of England and included all patients who were free from prior CVD (defined in **Supplementary Table 1**) on 1st January 2006. Risk factor measurements were included if recorded during two-year time window prior to this date, with preference given to measurements closest to baseline. Follow-up was to 31st December 2017. Incident non-fatal HF events obtained from Hospital Episode Statistics (recorded in any diagnostic position) and deaths from the Office for National Statistics data where HF was the underlying cause. Information on prior/prevalent events were supplemented by primary care records (using READCodes)
The HUNT3 Study[2]	Norway	The Trøndelag Health Study (HUNT) is a large population-based cohort study including extensive data from surveys, interviews, clinical measurements, and biological samples. All citizens > 20 years were invited (participation rate 54–89%), and four health surveys have been completed: HUNT1 (1984–86), HUNT2 (1995–97), HUNT3 (2006–08), and HUNT4 (2017–19). HUNT3 was used for external validation. In HUNT3, 93,000 people were invited, with 50,807 participating through questionnaires, interviews, clinical exams, and collection of blood and urine samples.
The Estonian Biobank, 2018 wave (EstBB)3	Estonia	The Estonian Biobank, run by the University of Tartu, expanded in 2018–2019 by recruiting approximately 150,000 new participants, increasing coverage to about 20% of Estonia's adult population. Participants provided broad consent and were enrolled through digitally streamlined procedures, including electronic signature via the national e-ID system and blood sample donation at a healthcare provider or pharmacy. Inclusion required adults able to provide informed consent. Data collected included baseline health questionnaires, clinical measurements, biological samples, and genome-wide genotypes, complemented by linkage to national health registries and electronic health records. This expansion also supported personalised medicine initiatives, such as the development of polygenic risk scores and pharmacogenetic recommendations.

1. Herrett E, Gallagher AM, Bhaskaran K, et al. Data Resource Profile: Clinical Practice Research Datalink (CPRD). Int J Epidemiol. 2015;44(3). doi:10.1093/ije/dyv098

2. Krokstad S, Langhammer A, Hveem K, et al. Cohort Profile: The HUNT Study, Norway. Int J Epidemiol. 2013;42(4):968-977. doi:10.1093/ije/dys095

3. Milani L, Alver M, Laur S, et al. The Estonian Biobank's journey from biobanking to personalized medicine. Nat Commun. 2025;16(1):3270. doi:10.1038/s41467-025-58465-3

Supplementary Table 5. Risk regions defined by age- and sex- standardised WHO CVD mortality rates per country (used in previous recalibration of the SCORE2 CVD risk models)

Country	Age and sex standardised CVD mortality per 100 000 person years, ICD chapter 9	Year collected
**Low risk region**		
France	70.9	2014
Israel	76.7	2015
Spain	89.4	2015
Netherlands	89.9	2016
Switzerland	90.2	2015
Denmark	90.4	2015
Norway	90.8	2015
Luxembourg	92.9	2015
Belgium	99.2	2015
United Kingdom	99.7	2015
**Moderate risk region**		
Iceland	101.0	2016
Portugal	107.9	2014
Sweden	109.0	2016
Italy	110.1	2015
San Marino	-	
Ireland	111.5	2014
Cyprus	111.5	2016
Finland	128.5	2015
Austria	130.9	2016
Malta	133.3	2015
Greece	138.8	2015
Germany	139.0	2015
Slovenia	143.3	2015

Country	Age and sex standardised CVD mortality per 100 000 person years, ICD chapter 9	Year collected
**High risk region**		
Albania	184.5	2010
Czech Republic	195.0	2016
Turkey	199.5	2015
Kazakhstan	214.0	2015
Croatia	214.6	2016
Poland	223.8	2015
Estonia	234.8	2015
Slovakia	239.2	2014
Hungary	274.1	2016
Bosnia and Herzegovina	279.2	2014
**Very high risk region**		
Armenia	306.3	2016
Lithuania	309.0	2016
Georgia	309.6	2015
Latvia	327.2	2015
Serbia	329.1	2015
Romania	330.5	2016
Montenegro	348.4	2009
Russian Federation	368.8	2015
TFYR Macedonia	387.8	2013
Belarus	395.4	2014
Azerbaijan	416.5	2007
Bulgaria	421.2	2014
Republic of Moldova	442.2	2016
Ukraine	476.7	2015
Kyrgyzstan	476.9	2015
Uzbekistan	478.6	2014
Egypt	543.7	2015
Morocco	-	
Syria	-	
Tunisia	-	
Lebanon	-	
Algeria	-	
Libya	-	

Countries without available population or incidence data in the WHO database (indicated by -) were grouped using rates available from neighbouring countries.

Supplementary Table 6. Characteristics of individuals contributing to the derivation cohorts for SCORE2-HF, by cohort

Cohort abbreviation	Country	Median year of baseline	Maximum year of follow up	Total individuals	Age at survey (yrs) mean (sd)	Sex, Men N (%)	Diabetes at baseline N (%)	Current smoking N (%)	Current use of anti-hypertensive medication N (%)	SBP (mmHg) mean (sd)	BMI (kg/m2) mean (sd)	eGFR** (ml/min/ 1.73m^2) mean (sd)	HbA1c (mmol/ mol) if diabetes mean(sd)	Glucose (mmol/l) mean (sd)	Age at diabetes diagnosis (yrs) mean(sd)	Incident Heart failure	Non-HF death	Median follow-up (5th & 95th percentiles)
BRUN	Italy	1990	2010	845	58 (11)	422 (50)	34 (4.0)	212 (25)	133 (16)	145 (21)	25 (4)	87 (14)	59 (20)	5.6 (1.1)	59 (13)	10	283	20.2 (4.9 to 20.5)
CASTEL	Italy	1984	1996	2654	74 (5)	1028 (39)	340 (12.8)	363 (14)	715 (27)	160 (25)	26 (4)	76 (17)	-	6.1 (1.7)	-	251	963	11.2 (2.3 to 14.0)
COPEN	Denmark	1993	2013	7194	63 (11)	3009 (42)	256 (3.6)	3533 (49)	831 (12)	142 (22)	26 (4)	71 (14)	-	5.9 (1.9)	56 (12)	132	3728	18.7 (2.7 to 21.1)
ESTHER	Germany	2001	2015	7962	61 (7)	3381 (42)	920 (11.9)	1372 (18)	2876 (37)	139 (20)	28 (4)	85 (20)	53 (15)	5.5 (1.6)	55 (10)	424	14	5.0 (1.0 to 5.9)
GOTOW	Sweden	1969	2009	1058	50 (4)	0 (0)	13 (1.2)	412 (39)	67 (6)	137 (23)	24 (4)	85 (15)	-	4.2 (0.9)	49 (9)	205	587	33.0 (10.2 to 41.0)
LIFE*	Scandinavia	1997	2001	799	67 (7)	350 (44)	72 (9.0)	132 (17)	0 (0)	174 (13)	28 (5)	80 (14)	-	4.2 (0.9)	-	12	50	4.5 (3.2 to 4.6)
MATISS83	Italy	1984	2002	2202	54 (8)	1042 (47)	119 (5.4)	635 (29)	0 (0)	142 (23)	28 (4)	91 (17)	-	5.3 (1.4)	-	60	416	18.7 (6.2 to 19.5)
MATISS87	Italy	1987	2002	1862	54 (8)	814 (44)	74 (4.0)	381 (21)	0 (0)	143 (22)	29 (4)	90 (17)	-	5.3 (1.2)	-	29	220	15.6 (7.4 to 16.2)
MPP	Sweden	1980	2010	24142	49 (5)	15116 (63)	921 (3.8)	10306 (44)	0 (0)	127 (16)	25 (4)	86 (14)	-	4.9 (1.0)	41 (11)	1470	5962	26.7 (8.4 to 34.3)
MRCOLD	UK	1996	2007	11899	81 (5)	4337 (36)	822 (6.9)	1405 (12)	3364 (29)	149 (22)	26 (4)	59 (15)	-	5.8 (2.1)	72 (10)	233	7613	7.9 (1.0 to 11.7)
PREVEND*	Netherlands	1998	2008	5649	54 (10)	2839 (50)	264 (4.7)	1907 (34)	971 (17)	132 (21)	27 (4)	84 (14)	-	5.0 (1.3)	56 (9)	192	426	10.6 (3.9 to 11.2)
PROSPER*	Scotland/Ireland /Netherland	1998	2002	3505	75 (3)	1497 (43)	425 (12.1)	1170 (33)	2527 (72)	157 (21)	27 (4)	61 (15)	-	5.5 (1.5)	-	100	282	3.3 (1.6 to 3.9)
RS_I	Netherlands	1992	2005	5658	69 (9)	2135 (38)	425 (7.7)	1243 (23)	1589 (28)	139 (22)	26 (4)	76 (15)	-	6.8 (2.5)	-	147	1950	11.9 (2.3 to 14.3)
SHIP	Germany	1999	2011	2636	58 (11)	1296 (49)	378 (14.4)	655 (25)	1003 (38)	142 (21)	28 (5)	81 (15)	55 (15)	5.9 (1.9)	54 (11)	15	431	11.3 (4.9 to 12.4)
ULSAM	Sweden	1972	2008	2302	50 (1)	2302 (100)	123 (5.4)	1167 (68)	90 (4)	133 (18)	25 (3)	99 (11)	-	5.0 (0.9)	48 (1)	418	1229	30.8 (8.8 to 37.8)
UKBIOBANK	UK	2009	2022	462914	57 (8)	203863 (44)	19580 (4.2)	47842 (10)	80220 (18)	138 (19)	27 (5)	95 (13)	55 (20)	5.1 (1.2)	51 (12)	13336	9208	13.5 (8.5 to 15.0)
ALLHAT*	USA/Canada/ Puerto Rico/US Virgin Islands	1994	2002	28861	66 (8)	14107 (49)	11652 (40.4)	7070 (24)	25751 (89)	146 (15)	30 (6)	76 (18)	-	7.0 (3.3)	-	1266	60	3.6 (0.2 to 6.4)
AUSDIAB	Australia	2000	2019	7770	56 (11)	3426 (44)	384 (4.9)	1094 (15)	1297 (17)	132 (19)	27 (5)	80 (13)	53 (19)	5.7 (1.2)	58 (13)	31	1636	19.0 (7.4 to 20.0)
ARIC	USA	1991	2017	12151	57 (6)	5461 (45)	1803 (14.9)	2985 (25)	3325 (28)	121 (19)	28 (5)	71 (12)	58 (23)	6.3 (2.3)	52 (10)	2358	3468	25.3 (5.6 to 27.6)
CHS1	USA	1989	2002	3997	72 (5)	1559 (39)	535 (13.4)	475 (12)	1487 (37)	136 (21)	26 (5)	69 (16)	76 (31)	6.0 (1.8)	59 (10)	765	1169	12.1 (2.3 to 12.9)
CHS2	USA	1993	2002	513	73 (6)	193 (38)	123 (24.0)	79 (15)	299 (58)	143 (24)	29 (6)	68 (18)	-	6.5 (2.9)	60 (11)	86	107	9.1 (1.4 to 9.5)
DHS	USA	2002	2013	1639	51 (7)	717 (44)	216 (14.6)	80 (6)	188 (16)	130 (19)	30 (7)	94 (16)	57 (20)	5.7 (2.1)	-	34	81	11.8 (4.8 to 13.1)
FRAMOFF	USA	1996	2008	3059	58 (9)	1372 (45)	255 (8.3)	61 (2)	865 (28)	128 (19)	28 (5)	65 (14)	-	5.7 (1.4)	-	101	289	11.8 (5.7 to 13.7)
NHANESIII	USA	1990	2013	9642	61 (14)	4449 (46)	1006 (10.4)	2347 (24)	2227 (65)	134 (21)	27 (5)	69 (17)	63 (23)	5.9 (2.3)	54 (14)	119	4620	17.8 (2.4 to 22.3)
RANCHO	USA	1998	2009	865	73 (11)	291 (34)	57 (6.6)	36 (4)	307 (36)	135 (21)	26 (4)	75 (17)	-	5.6 (1.1)	-	24	199	8.7 (1.7 to 10.5)

***** Data source is a clinical trial

****** eGFR: estimated Glomerular Filtration Rate (ml/min/1.73m^2) calculated using the CKD epi 2021 equations

Supplementary Table 7: Subdistribution hazard ratios from the SCORE-HF risk models

**Centred predictors included in the Core model**	**SHR (95% CI)**	
	**Men**<br>(N = 270,222, HF events =12,161	**Women**<br>(N = 330,483, HF events = 9556)
Predictors used for all individuals		
Age (per 5 years)	1.58 (1.55, 1.62)	1.70 (1.66, 1.74)
Current smoking	1.64 (1.57, 1.72)	2.06 (1.94, 2.18)
SBP (per SD: 20 mmHg)	1.16 (1.13, 1.19)	1.16 (1.13, 1.19)
History of diabetes	1.16 (1.07, 1.26)	1.31 (1.16, 1.47)
Antihypertensive medication use	1.59 (1.48, 1.70)	1.72 (1.60, 1.84)
BMI (per SD: 5 kg/m^2)	1.36 (1.31, 1.40)	1.36 (1.33, 1.40)
BMI^2	1.00 (0.99, 1.01)	0.99 (0.99, 1.00)
Ln eGFR* (per SD: 0.15)	0.97 (0.95, 0.99)	0.95 (0.93, 0.97)
Ln eGFR^2	1.00 (1.00, 1.01)	1.00 (1.00, 1.01)
SBP * Hypertension drug use	0.96 (0.92, 1.00)	0.95 (0.91, 0.99)
Age * Current smoking	0.94 (0.92, 0.96)	0.90 (0.87, 0.93)
Age * SBP	0.98 (0.97, 0.99)	0.99 (0.98, 1.00)
Age * History of diabetes	1.03 (0.97, 1.10)	1.01 (0.92, 1.12)
Age * Hypertension drug use	0.97 (0.94, 0.99)	0.95 (0.92, 0.98)
Age * BMI	0.98 (0.97, 1.00)	0.97 (0.96, 0.98)
Age * Ln eGFR	1.00 (1.00, 1.01)	1.00 (1.00, 1.01)
Predictors used for individuals with diabetes		
Age of diabetes diagnosis (per 5 years)	0.97 (0.95, 1.00)	0.91 (0.87, 0.96)
Hba1c (per SD: 9.3 mmol/mol)	1.11 (1.09, 1.13)	1.13 (1.09, 1.16)
Age*HbA1c	0.98 (0.97, 1.00)	0.99 (0.96, 1.02)

Baseline survival at 10 years for covariates at zero values for Women= 0.9923, Men= 0.9885

eGFR: estimated Glomerular Filtration Rate (ml/min/1.73m^2) calculated using the CKD epi 2021 equations

Models derived in UK Biobank and 24 cohorts from the Emerging Risk Factors Collaboration, using sex-specific Fine and Gray models stratified by cohort.

Supplementary Table 8. Recalibration factors to recalibrate risk estimates from the core models to region-specific 10-year or 30-year risk

		10-year recalibration factors		30-year recalibration factors	
Risk region	Sex	Scale 1	Scale 2	Scale 1	Scale 2
Low	Men	0.2247	0.8758	1.2857	0.6971
Low	Women	-0.0229	0.8181	1.6742	0.7311
Moderate	Men	0.5441	0.8695	1.5053	0.6786
Moderate	Women	0.3562	0.8511	2.1173	0.7724
High	Men	0.707	0.7344	0.7495	0.3944
High	Women	0.7611	0.7779	1.6327	0.5536
Very High	Men	1.0927	0.6785	0.965	0.3275
Very High	Women	1.264	0.7459	1.7426	0.4598

Supplementary Table 9: Summary of data used in external validation of the SCORE2-HF models

	Mean (SD), Median [IQR] or n(%)		
	**CPRD**	**HUNT3**	**Estonian Biobank**
Number of participants with no History of CVD or HF at baseline	1,227,276	33,434	76,114
Men	588,246 (48)	14,931 (45)	25,001 (33)
Predictors included in SCORE2-HF			
Age (years)	55 [48, 66]	57 [49, 65]	52 [45, 60]
Systolic Blood Pressure (SBP, mmHg)	134 (17)	133 (19)	130 (18)
Body Mass Index (BMI, kg/m^2)	27.6 (5.4)	27.4 (4.3)	26.9 (5.1)
eGFR* (ml/min/1.73^2)	80 [68, 94]	95 (16)	89 (15)
Current hypertensive medication use	362,591 (30)	7,285 (22)	21917 (29)
Current smoking	501353 (41)	5,930 (18)	9466 (12)
History of diabetes	62954 (5)	1,553 (4.6)	4730 (6.2)
Predictors used only in those with diabetes			
Hba1c (mmol/mol)	40 [34, 47]	52 [44, 63]	45 [41, 56]
Age of diabetes diagnosis (years)	59 [50, 67]	55 [46, 63]	50 [41, 59]
Incident events and follow-up			
Heart failure	33,946 (3)	2054 (6.1)	841 (1.1)
Follow-up (years)	8.2 [4.9, 10.2]	15.9 [15.4, 16.4]	4.9 [0.9, 5.1]
Estimated 10-yr risk using each HF risk score			
Risk according to SCORE2-HF	2.4 [1.2, 5.5]	2.4 [1.1, 5.1]	6.7 [3.1, 13.4]
Risk according to PREVENT-HF	3.0 [1.3, 7.4]	2.4 [1.0, 5.9]	2.4 [0.9, 5.9]

*Estimated Glomerular Filtration rate, estimated using the 2021 CKD creatinine-based equations
CPDR= Clinical Practice Research Datalink; HUNT3 = Survey 3 of The Trøndelag Health Study; ; EstBB= Estonian Biobank

Supplementary Figure 1: Selection of participants contributing to studies involved in SCORE2-HF model derivation and external validation

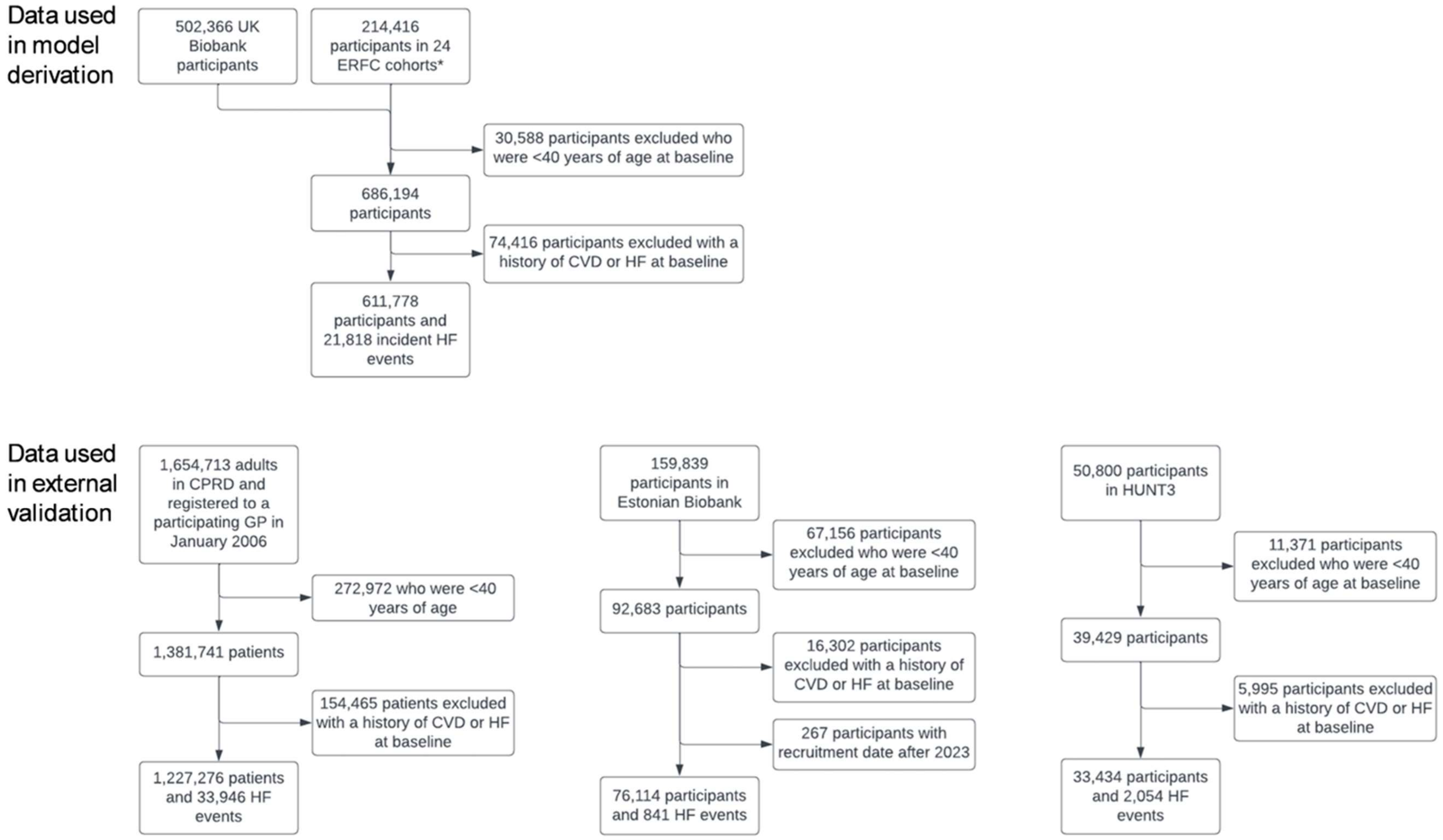

ERFC= Emerging Risk Factors Collaboration; CPDR= Clinical Practice Research Datalink; HUNT3 = Survey 3 of The Trøndelag Health Study
*Cohort level inclusion criteria for the ERFC: population-based studies that had recorded cause-specific deaths and/or non-fatal HF events for at least 1-year of follow-up; and had recorded baseline information on age, current smoking, history of type-2 diabetes mellitus (T2DM), systolic blood pressure (SBP), antihypertensive medication use, body mass index (BMI), estimated glomerular filtration rate (eGFR, estimated using the 2021 CKD EPI creatinine-based equations27) or creatinine (to calculate eGFR), and T2DM characteristics (age at diagnosis and HbA1c, or glucose).

Supplementary Figure 2: Selection of participants contributing to data sources involved in SCORE2-HF model recalibration

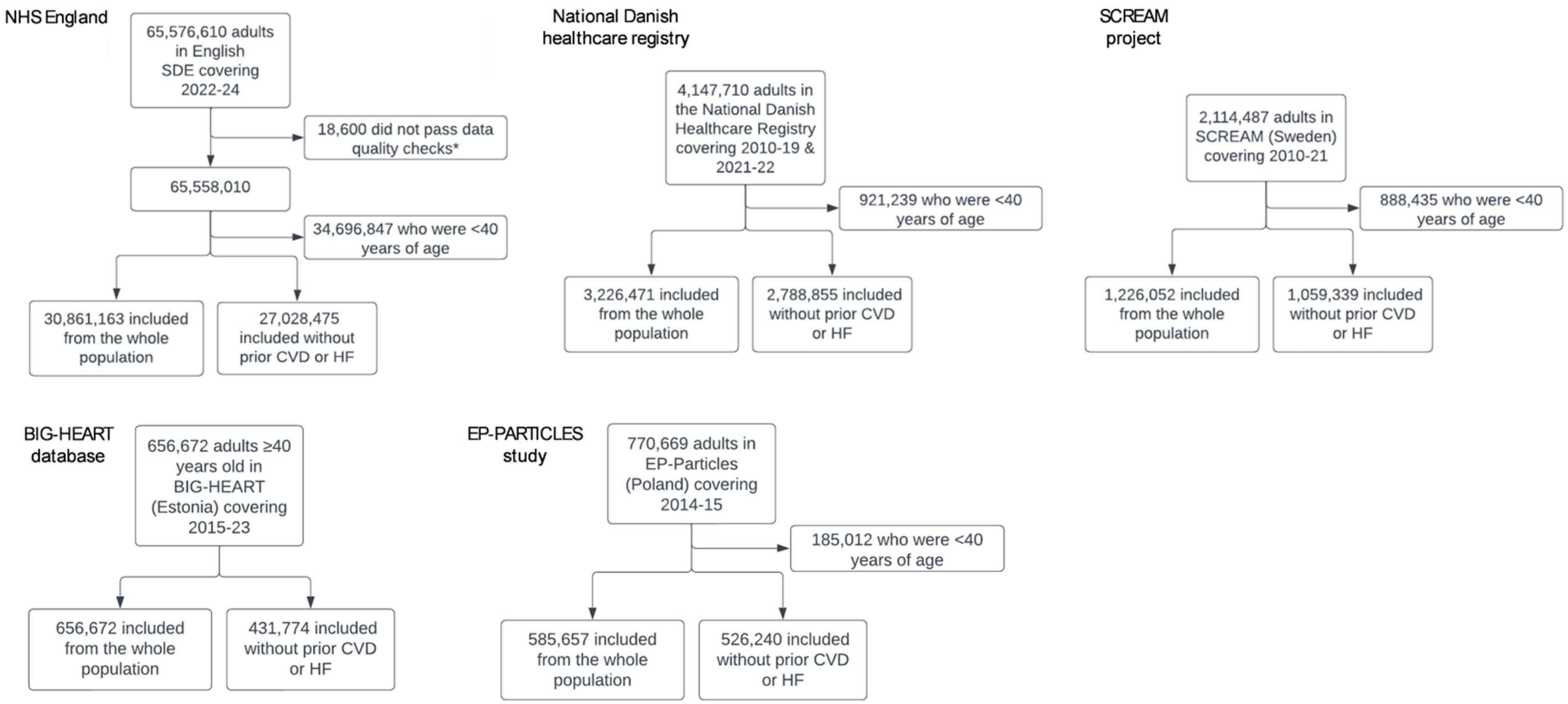

Flow of patients/participants in data sources used for multiplier calculation. Numbers represent the average annual numbers covering the years shown in the top node for each data source. Further information on each data source is given in Supplementary Tables 2 and 3

SDE= Secure Data Environment, SCREAM project = The Stockholm CREAtinine Measurements (SCREAM) project

*Reasons for not passing data quality checks in NHS England included: 1) The year of birth is after the year of death; 2) Patient does not have mandatory fields completed (NHS_number, sex, Date of birth); 3) Year of Birth Predates NHS Established Year or Year is over the Current Date; 4) Remove those with only null/invalid dates of death; 5) Remove those where the registered date of death is before the actual date of death; 6) Pregnancy/birth codes for Men; 7) Prostate Cancer Codes for Women; 8) Patients have all missing record dates and dates

Supplementary Figure 3: Sub-distribution hazard ratios for the SCORE2-HF models; change across levels of each risk predictor and change with age

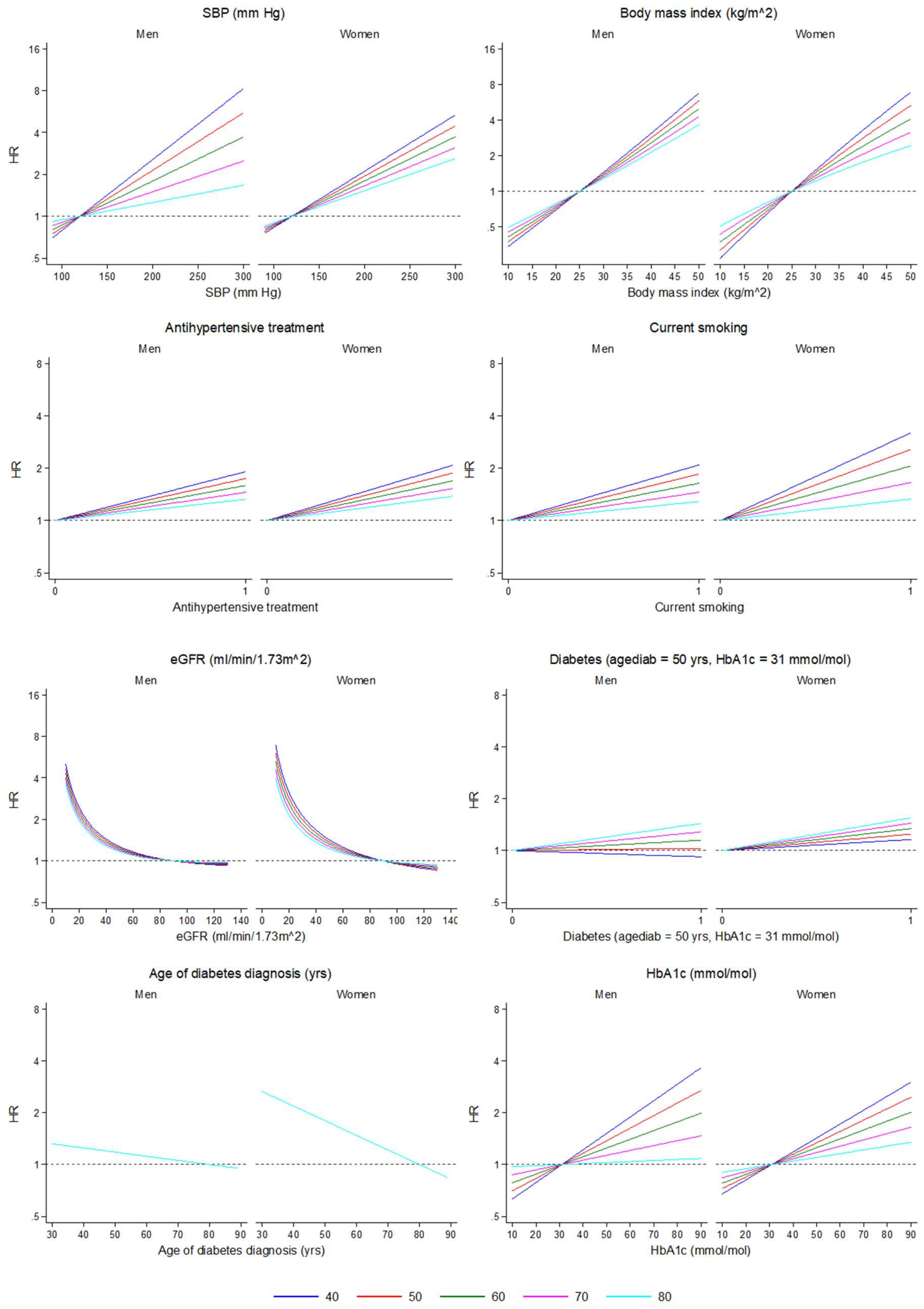

Agediab = age of diabetes diagnosis.

Supplementary Figure 4: Multipliers used to convert WHO mortality statistics into HF and non-HF death statistics, by data source

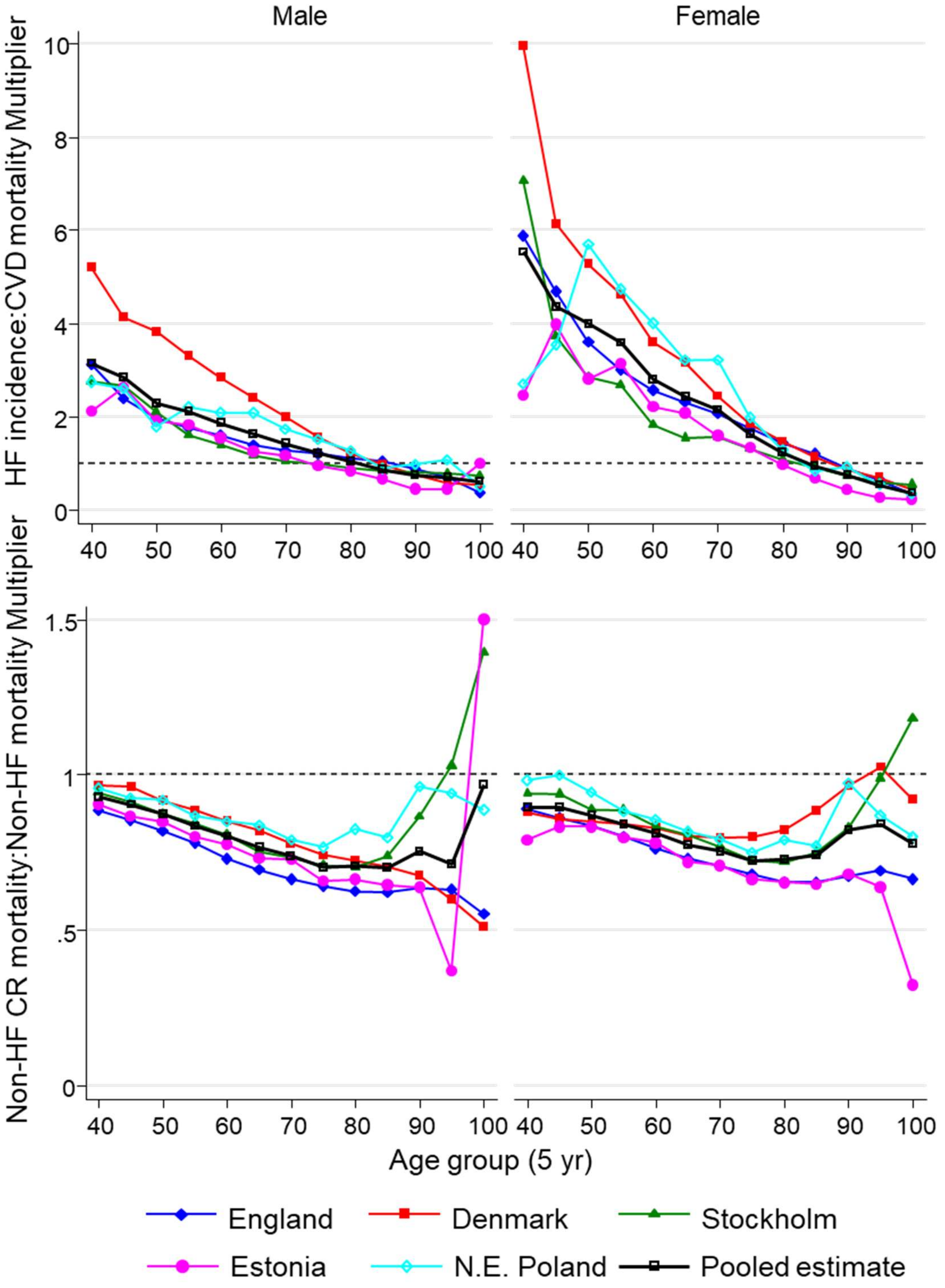

Non-HF CR mortality: Non-Heart failure competing risk mortality

Supplementary Figure 5: Incidence rates used to recalibrate the models, and risks estimated by the SCORE2-HF models before and after recalibration, by sex, region and 5-year age group

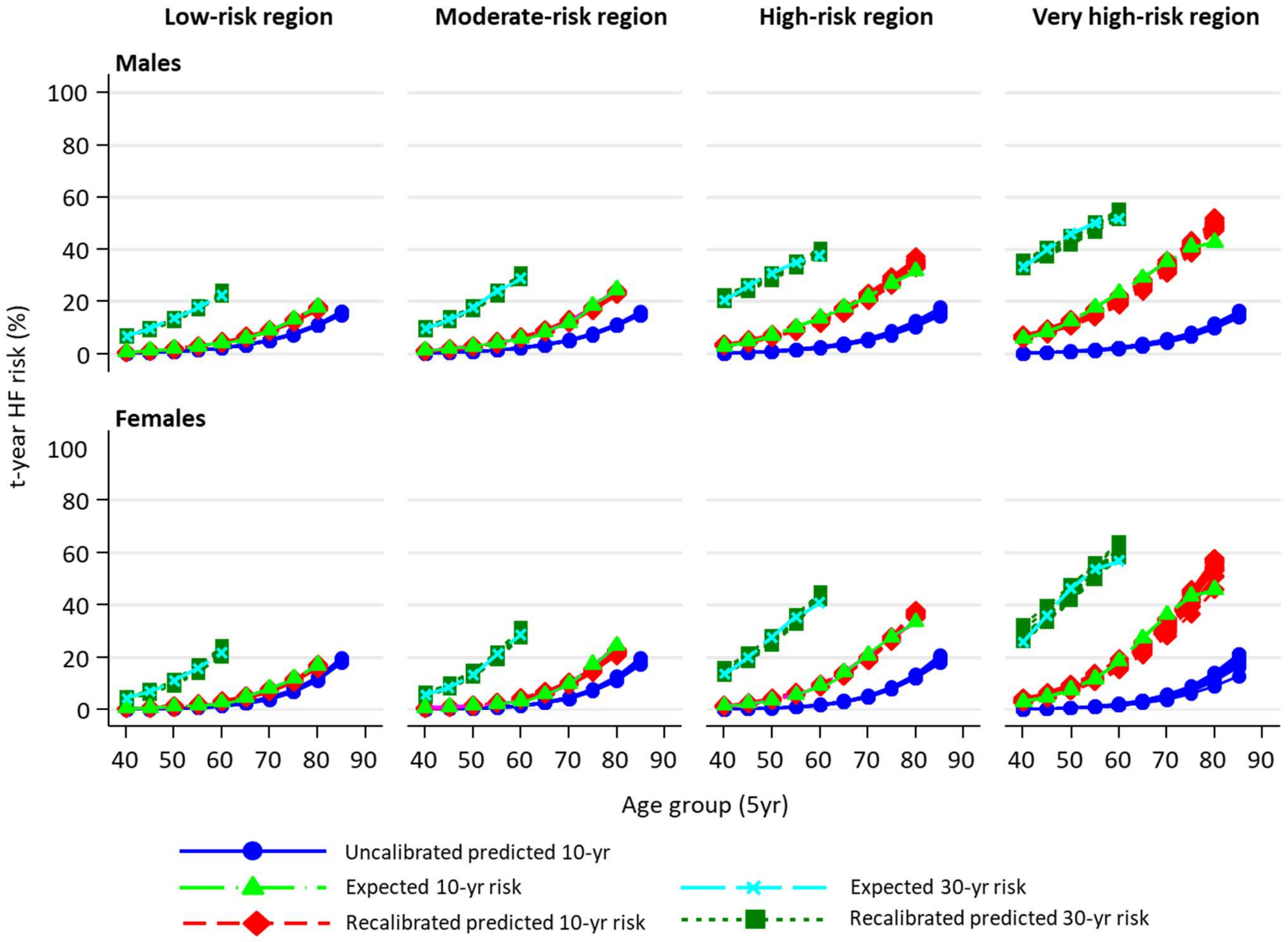

Individual uncalibrated and recalibrated lines represent risks estimated using country specific risk factors from different European countries situated in each risk region. Predicted and recalibrated risks are estimated using the SCORE2-HF models, with and without recalibration to the estimated expected average level of risk in each whole risk region.

Supplementary Figure 6. C-index results from internal and external validation, by sex

Sex	Validation	Study	Individuals	Events	Risk score	C-index (95% CI)	Difference in C-index (95% CI) PREVENT-HF – SCORE2-HF
**Men**	*Internal validation*	UKB/ERFC	275,006	12,201	SCORE2-HF	0.728 (0.722, 0.733)	ref
					PREVENT-HF	0.722 (0.716, 0.727)	-0.006 (-0.008, -0.004)
	External validation	CPRD	588,246	16,563	SCORE2-HF	0.808 (0.805, 0.812)	ref
					PREVENT-HF	0.804 (0.801, 0.808)	-0.004 (-0.005, -0.003)
		HUNT3	14,931	502	SCORE2-HF	0.819 (0.801, 0.836)	ref
					PREVENT-HF	0.820 (0.803, 0.837)	0.001 (-0.003, 0.005)
		EstBB	25,001	375	SCORE2-HF	0.845 (0.827, 0.862)	ref
					PREVENT-HF	0.840 (0.823, 0.858)	-0.004 (-0.010, 0.001)
**Women**	*Internal validation*	UKB/ERFC	336,772	9,617	SCORE2-HF	0.763 (0.757, 0.769)	ref
					PREVENT-HF	0.756 (0.750, 0.762)	-0.007 (-0.008, -0.005)
	External validation	CPRD	639,030	17,383	SCORE2-HF	0.843 (0.840, 0.846)	ref
					PREVENT-HF	0.842 (0.839, 0.845)	-0.001 (-0.002, -0.000)
		HUNT3	18,503	537	SCORE2-HF	0.855 (0.839, 0.870)	ref
					PREVENT-HF	0.857 (0.843, 0.872)	0.003 (-0.000, 0.006)
		EstBB	51,113	466	SCORE2-HF	0.886 (0.872, 0.900)	ref
					PREVENT-HF	0.886 (0.871, 0.900)	-0.000 (-0.004, 0.003)

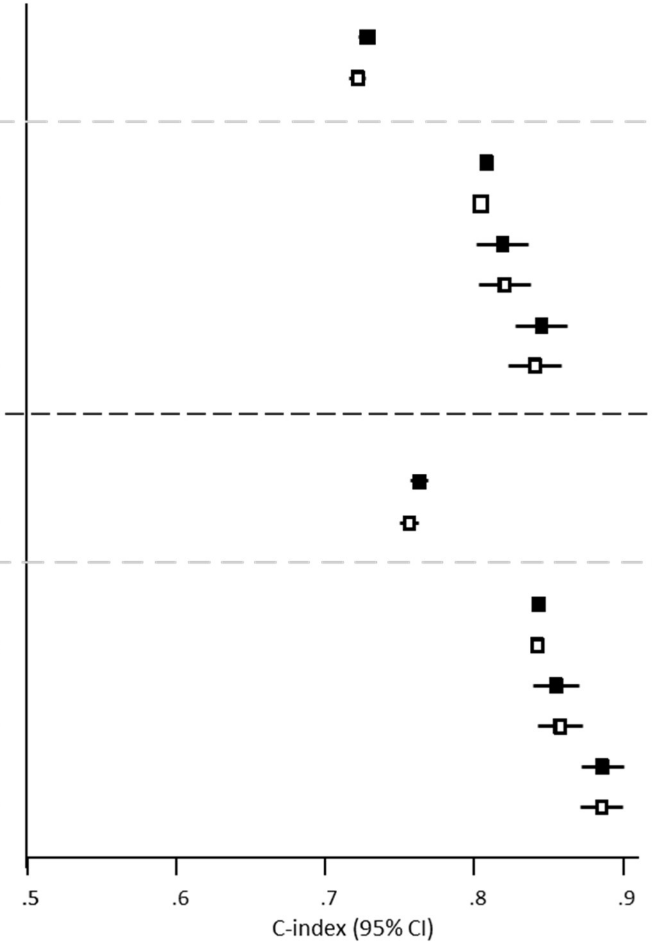

UKB= UK Biobank, ERFC= Emerging Risk Factors Collaboration; CPDR= Clinical Practice Research Datalink; HUNT3 = Survey 3 of The Trøndelag Health Study; EstBB= Estonian Biobank

Supplementary Figure 7. C-index results from internal and external validation, by age group

		Study	Individuals	Events	Risk score	C-index (95% CI)	Difference in C-index (95% CI) PREVENT-HF – SCORE2-HF
**Individuals aged 40-59**	*Internal validation*	UKB/ERFC	342,350	7,690	SCORE2-HF	0.725 (0.717, 0.733)	ref
					PREVENT-HF	0.715 (0.707, 0.723)	-0.010 (-0.013, -0.007)
	External validation	CPRD	773,306	6,398	SCORE2-HF	0.752 (0.743, 0.761)	ref
					PREVENT-HF	0.737 (0.729, 0.745)	-0.015 (-0.019, -0.011)
		HUNT3	20182	149	SCORE2-HF	0.721 (0.682, 0.760)	ref
					PREVENT-HF	0.733 (0.695, 0.770)	0.011 (-0.003, 0.026)
		EstBB	55,609	203	SCORE2-HF	0.837 (0.809, 0.866)	ref
					PREVENT-HF	0.833 (0.804, 0.862)	-0.005 (-0.017, 0.007)
**Individuals aged 60+**	*Internal validation*	UKB/ERFC	269,428	14,128	SCORE2-HF	0.684 (0.679, 0.690)	ref
					PREVENT-HF	0.676 (0.670, 0.681)	-0.009 (-0.011, -0.007)
	External validation	CPRD	453,970	27,548	SCORE2-HF	0.727 (0.724, 0.730)	ref
					PREVENT-HF	0.727 (0.723, 0.730)	-0.000 (-0.002, 0.001)
		HUNT3	13,252	890	SCORE2-HF	0.750 (0.735, 0.766)	ref
					PREVENT-HF	0.752 (0.737, 0.768)	0.002 (-0.002, 0.006)
		EstBB	20,505	638	SCORE2-HF	0.768 (0.750, 0.786)	ref
					PREVENT-HF	0.766 (0.748, 0.785)	-0.002 (-0.008, 0.004)

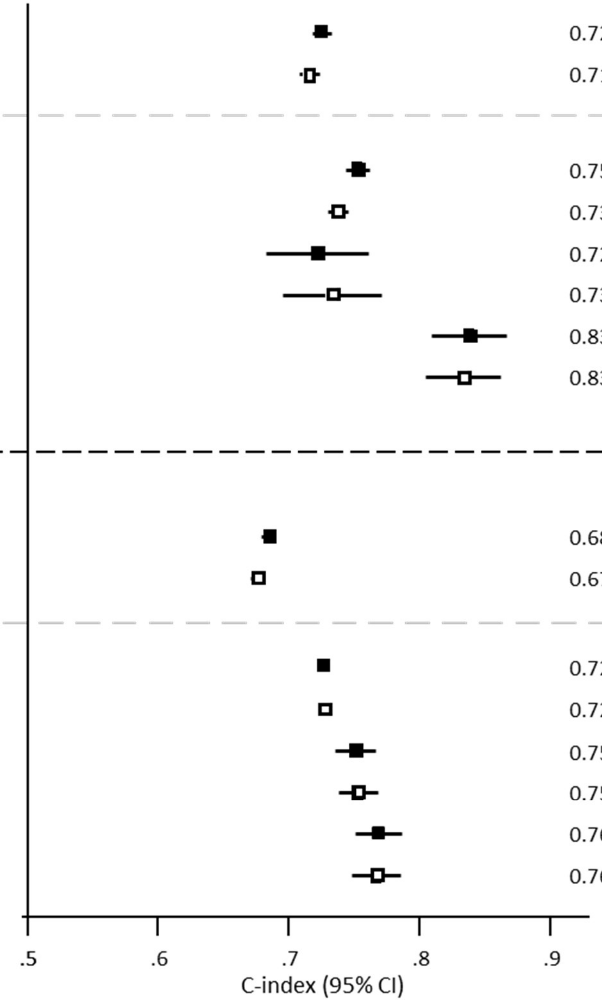

UKB= UK Biobank, ERFC= Emerging Risk Factors Collaboration; CPDR= Clinical Practice Research Datalink; HUNT3 = Survey 3 of The Trøndelag Health Study; EstBB= Estonian Biobank

Supplementary Figure 8. C-index results from internal and external validation, in individuals with and without a history of T2DM

		Study	Individuals	Events	Risk score	C-index (95% CI)	Difference in C-index (95% CI) PREVENT-HF – SCORE2-HF
**Individuals without T2DM**	Internal validation	UKB/ERFC	570,765	18,293	SCORE2-HF	0.745 (0.741, 0.749)	ref
					PREVENT-HF	0.739 (0.734, 0.743)	-0.006 (-0.007, -0.005)
	External validation	CPRD	116,4322	28,718	SCORE2-HF	0.825 (0.822, 0.828)	ref
					PREVENT-HF	0.822 (0.820, 0.825)	-0.003 (-0.004, -0.002)
		HUNT3	31,881	919	SCORE2-HF	0.838 (0.826, 0.851)	ref
					PREVENT-HF	0.841 (0.829, 0.853)	0.003 (0.000, 0.006)
		EstBB	71,384	667	SCORE2-HF	0.872 (0.860, 0.884)	ref
					PREVENT-HF	0.870 (0.857, 0.882)	-0.002 (-0.006, 0.001)
**Individuals with T2DM**	Internal validation	UKB/ERFC	41,013	3,525	SCORE2-HF	0.690 (0.679, 0.701)	ref
					PREVENT-HF	0.680 (0.669, 0.691)	-0.014 (-0.021, -0.007)
	External validation	CPRD	62,954	5,228	SCORE2-HF	0.725 (0.718, 0.732)	ref
					PREVENT-HF	0.718 (0.712, 0.725)	-0.007 (-0.010, -0.003)
		HUNT3	1,553	120	SCORE2-HF	0.777 (0.739, 0.814)	ref
					PREVENT-HF	0.780 (0.744, 0.816)	0.003 (-0.012, 0.019)
		EstBB	4,730	174	SCORE2-HF	0.788 (0.756, 0.821)	ref
					PREVENT-HF	0.780 (0.748, 0.812)	-0.008 (-0.019, 0.003)

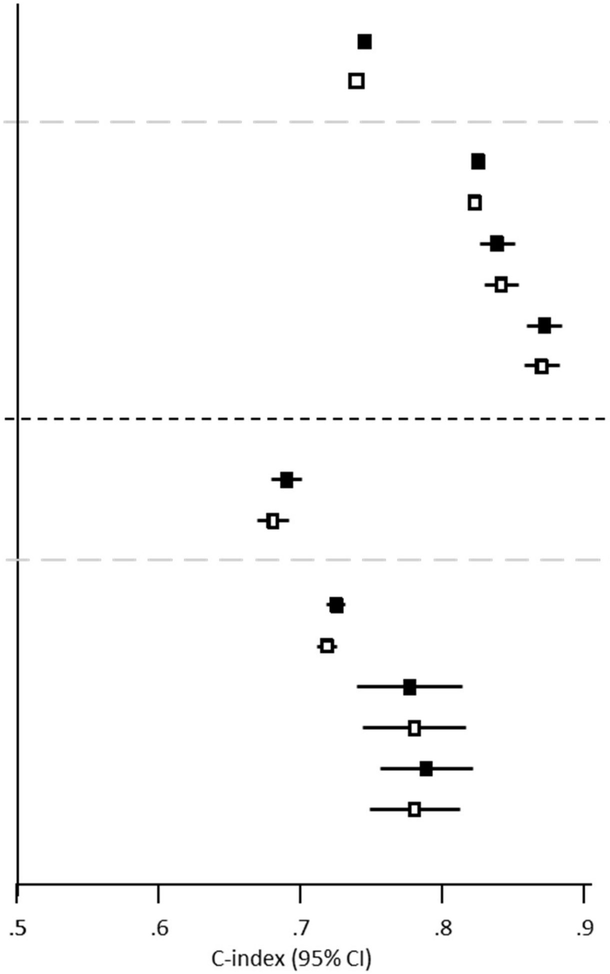

UKB= UK Biobank, ERFC= Emerging Risk Factors Collaboration; CPDR= Clinical Practice Research Datalink; HUNT3 = Survey 3 of The Trøndelag Health Study; EstBB= Estonian Biobank

Supplementary Figure 9. C-index for 30-year risks using the SCORE2-HF models among individuals ages 40-59 years

Study	Individuals	Events	Risk score	C-index (95% CI)	Difference in C-index (95% CI) PREVENT-HF – SCORE2-HF
Internal validation					
UKB/ERFC	342,350	7,690	SCORE2-HF	0.718 (0.711, 0.726)	ref
			PREVENT-HF	0.713 (0.706, 0.721)	-0.005 (-0.008, -0.002)
External validation					
CPRD	773,306	6,398	SCORE2-HF	0.741 (0.732, 0.750)	ref
			PREVENT-HF	0.737 (0.729, 0.745)	-0.004 (-0.008, -0.000)
HUNT3	20,182	391	SCORE2-HF	0.706 (0.681, 0.732)	ref
			PREVENT-HF	0.714 (0.689, 0.738)	0.007 (-0.003, 0.018)
EstBB	55,609	203	SCORE2-HF	0.827 (0.800, 0.855)	ref
			PREVENT-HF	0.834 (0.806, 0.863)	0.007 (-0.004, 0.018)

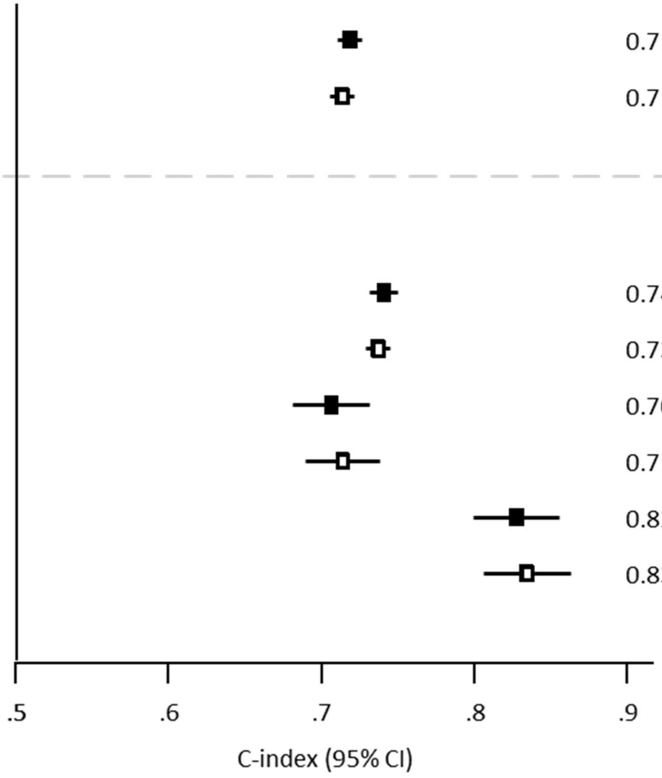

UKB= UK Biobank, ERFC= Emerging Risk Factors Collaboration; CPDR= Clinical Practice Research Datalink; HUNT3 = Survey 3 of The Trøndelag Health Study; EstBB= Estonian Biobank

Supplementary Figure 10: Calibration of the SCORE2-HF and PREVENT-HF models in CPRD

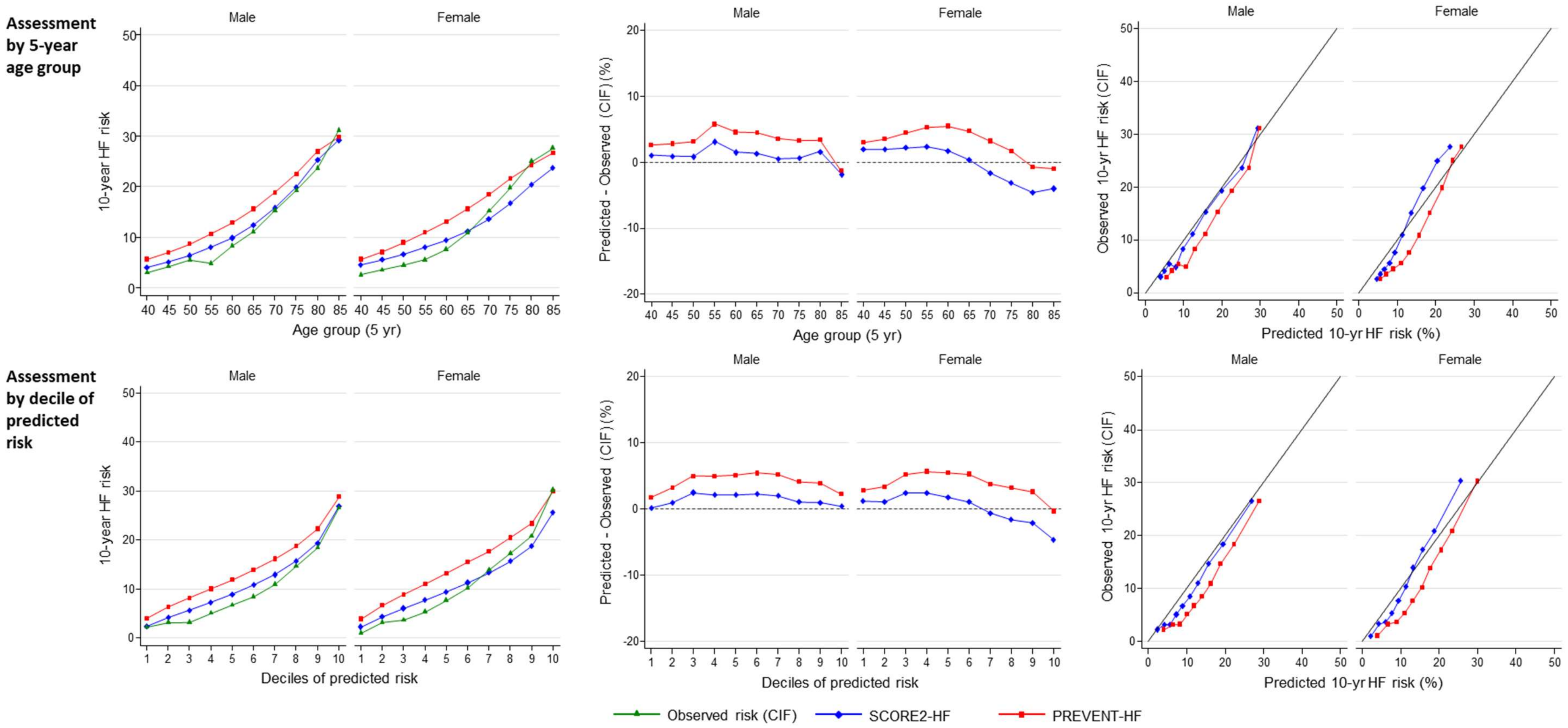

Among those individuals in CPRD who had an observed HF event within 10-years (median [IQR] follow-up = 8.2 [4.9, 10.2]), 8% had an MI or stroke prior to their HF event.

	Male		Female	
Calibration statistic	SCORE2-HF	PREVENT-HF	SCORE2-HF	PREVENT-HF
Intercept	-0.13 (-0.15, -0.11)	-0.31 (-0.32, -0.29)	-0.19 (-0.21, -0.17)	-0.38 (-0.40, -0.36)
Slope	1.24 (1.21, 1.27)	1.16 (1.13, 1.19)	1.28 (1.25, 1.31)	1.19 (1.16, 1.21)
O/E ratio	0.88 (0.86, 0.89)	0.74 (0.72, 0.75)	0.83 (0.81, 0.84)	0.68 (0.67, 0.70)

Supplementary Figure 11: Calibration of the SCORE2-HF and PREVENT-HF models in individuals with Type 2 diabetes from CPRD

Calibration assessed by 5-year age group

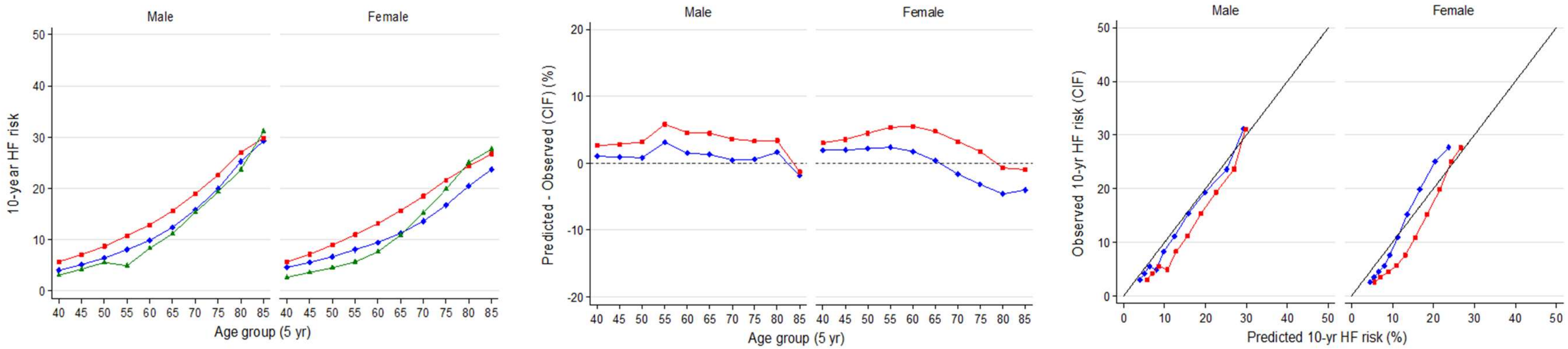

Calibration assessed by decile of predicted risk

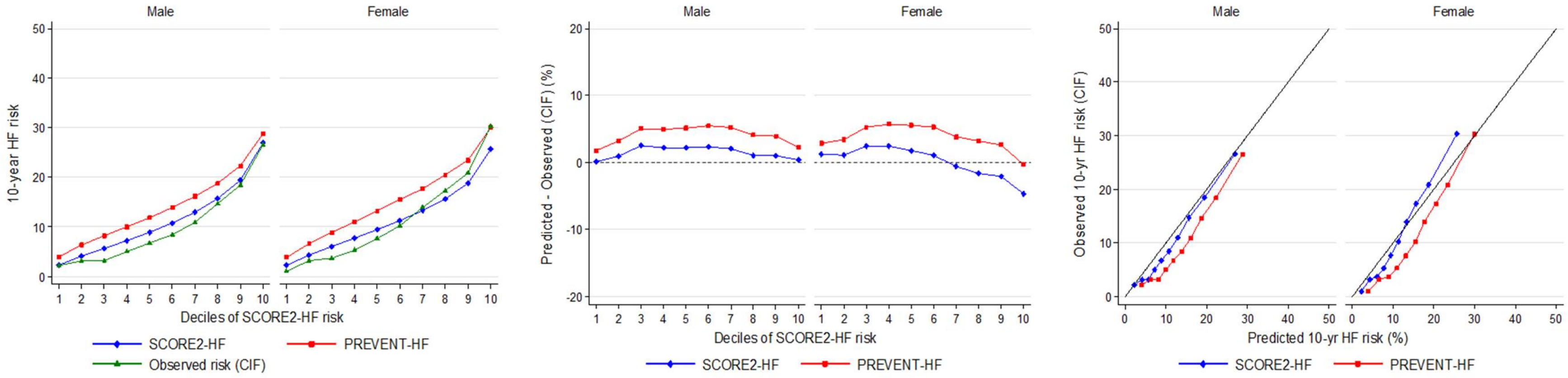

Group	Male		Female	
Calibration statistic	SCORE2-HF	PREVENT-HF	SCORE2-HF	PREVENT-HF
Intercept	-0.06 (-0.11, -0.02)	-0.28 (-0.33, -0.23)	0.07 (0.03, 0.12)	-0.24 (-0.29, -0.20)
Slope	1.16 (1.07, 1.24)	1.22 (1.12, 1.31)	1.23 (1.14, 1.32)	1.17 (1.08, 1.26)
O/E ratio	0.94 (0.90, 0.98)	0.76 (0.72, 0.79)	1.08 (1.03, 1.12)	0.78 (0.75, 0.82)

Supplementary Figure 12: Estimated risk distributions using SCORE2-HF in each risk region of Europe, by sex and 5-year age group

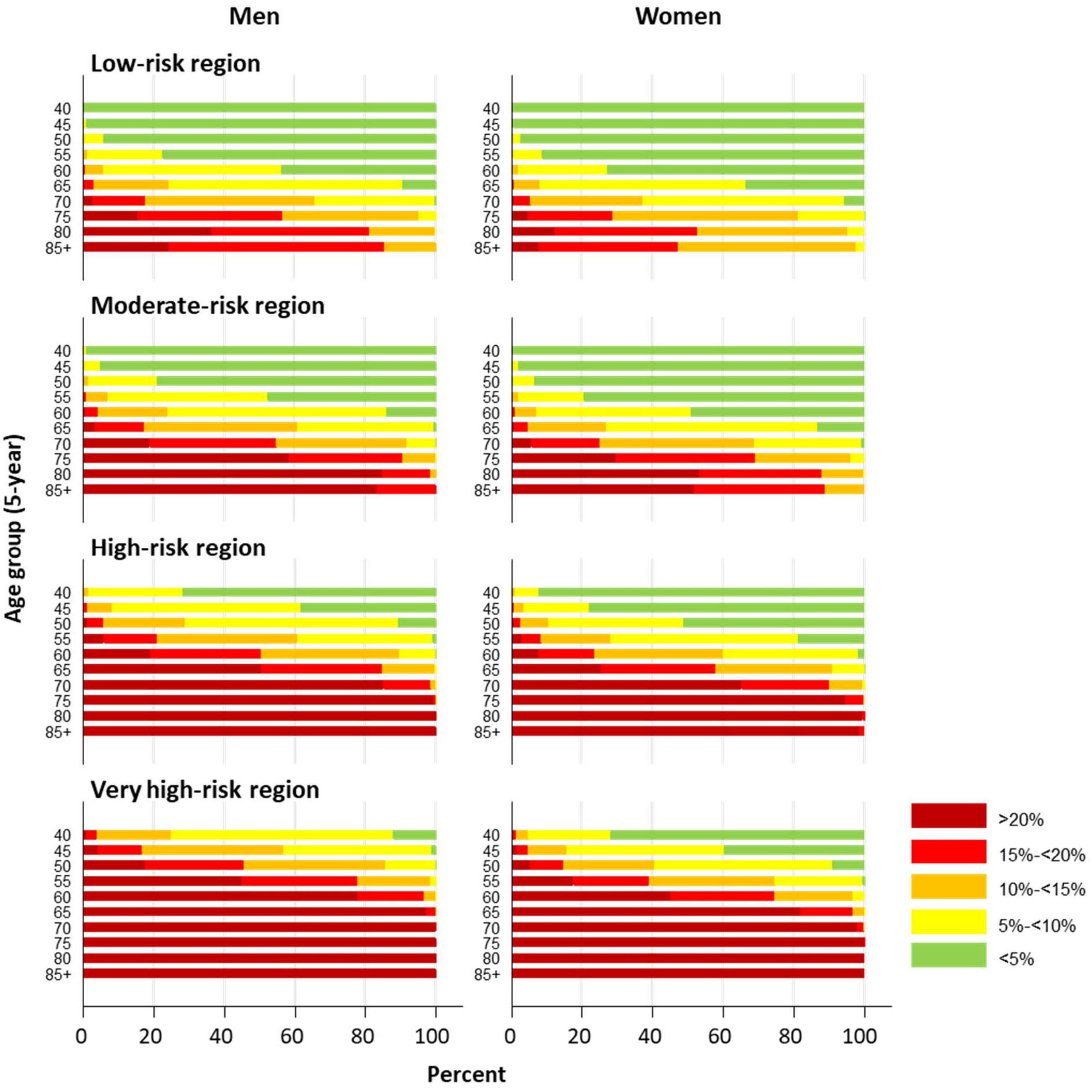

Funding sources for individual studies contributing to this analysis

This **Cardiovascular Health Study** research was supported by NHLBI contracts HHSN268201200036C, HHSN268200800007C, HHSN268201800001C, N01HC55222, N01HC85079, N01HC85080, N01HC85081, N01HC85082, N01HC85083, N01HC85086, 75N92021D00006; and NHLBI grants U01HL080295, R01HL087652, R01HL103612, R01HL105756, R01HL120393, U01HL130114, and R01HL172803 with additional contribution from the National Institute of Neurological Disorders and Stroke (NINDS). Additional support was provided through R01AG023629 from the National Institute on Aging (NIA). A full list of principal CHS investigators and institutions can be found at CHS-NHLBI.org.

EP-PARTICLES study: This work was supported by the Polish National Agency for Academic Exchange (NAWA) in collaboration with the Medical Research Agency (ABM), grant no. BPN/WAL/2024/1/00010/U/00001, and by the Medical University of Białystok under grant no. B.SUB.25.560.

The BIG-HEART study was supported by the Estonian Research Council grants PSG809 and PRG1844, and the Estonian Ministry of Education and Research (project TEM-TA72; and grant TK218 establishing the Estonian Centre of Excellence for
